# Attenuated Oral Typhoid Vaccine Ty21a Elicits Lamina Propria and Intra-Epithelial Lymphocyte Tissue-Resident Effector Memory CD8 T Responses in the Human Terminal Ileum

**DOI:** 10.3389/fimmu.2019.00424

**Published:** 2019-03-14

**Authors:** Jayaum S. Booth, Seema A. Patil, Eric Goldberg, Robin S. Barnes, Bruce D. Greenwald, Marcelo B. Sztein

**Affiliations:** ^1^Center for Vaccine Development and Global Health, University of Maryland School of Medicine, Baltimore, MD, United States; ^2^Department of Pediatrics, University of Maryland School of Medicine, Baltimore, MD, United States; ^3^Department of Medicine, University of Maryland School of Medicine, Baltimore, MD, United States; ^4^Division of Gastroenterology and Hepatology, University of Maryland School of Medicine, Baltimore, MD, United States

**Keywords:** terminal ileum, *S*. Typhi, tissue resident memory CD8+ T, LPMC, lamina propria mononuclear cells, IEL intraepithelial lymphocytes, mucosal immune responses

## Abstract

Tissue-resident memory T cells (T_RM_) are newly defined memory T cells (T_M_) distinct from circulating T_M_ subsets which have the potential to mount rapid protective immune responses at the site of infection. However, very limited information is available regarding the role and contribution of T_RM_ in vaccine-mediated immune responses in humans at the site of infection. Here, we studied the role and contribution of tissue resident memory T cells (T_RM_) located in the terminal ileum (TI) (favored site of infection for *S*. Typhi) following oral Ty21a immunization in humans. We examined TI-lamina propria mononuclear cells (LPMC) and intra-epithelial lymphocytes (IEL) CD8+ T_RM_ subsets obtained from healthy volunteers undergoing medically-indicated colonoscopies who were either immunized with Ty21a or unvaccinated. No significant differences in the frequencies of LPMC CD8+ T_RM_ and CD8+CD69+CD103– T cells subsets were observed following Ty21a-immunization. However, LPMC CD8+ T_RM_ exhibited significantly higher levels of cytokines (IFN-γ, IL-17A, and TNF-α) *ex-vivo* in Ty21a-vaccinated than in unvaccinated volunteers. LPMC CD8+ T_RM_ *S*. Typhi-specific responses were evaluated using *S*. Typhi-infected targets and found to produce significantly higher levels of *S*. Typhi-specific IL-17A. In contrast, LPMC CD8+CD69+CD103- T cells produced significantly increased *S*. Typhi-specific levels of IFN-γ, IL-2, and IL-17A. Finally, we assessed CD8+ T_RM_ in IEL and observed that the frequency of IEL CD8+ T_RM_ is significantly lower following Ty21a immunization. However, *ex-vivo* IEL CD8+ T_RM_ elicited by Ty21a immunization spontaneously produced significantly higher levels of cytokines (IFN-γ, IL-17A, IL-2, and TNF-α). This study provides the first demonstration of the effect of oral Ty21a vaccination on CD8+ T_RM_ subsets (spontaneous and *S*. Typhi-specific) responses in the LPMC and IEL compartment of the human terminal ileum mucosa, contributing novel information to our understanding of the generation of mucosal immune responses following oral Ty21a-immunization.

## Introduction

Immunological memory is essential for vaccine protection and efficacy and for maintaining long-term immunity following exposure to wild-type pathogens. It is now becoming clear that protective immunity relies on circulating T memory (T_M_) cells, as well as non-circulating T_M_ which are abundant in peripheral tissues, especially in mucosal sites (1, 2). Tissue resident memory T cells (T_RM_) is a newly defined subset of T_M_ which is phenotypically distinct from circulating T_M_ subsets (e.g., T central/memory—T_CM_, T effector/memory—T_EM_, and CD45RA+ T_EM_–T_EMRA_). After induction, T_RM_ persist for a long time in peripheral tissues (particularly intestinal tissues) and represent a non-migratory population of T_M_ which has the potential to mediate rapid protective antigen recall responses (2). T_RM_ in the human gut are phenotypically characterized by their expression of CD69 (an activation and tissue retention marker via the sequestration of the sphingosine-1-phosphate receptor (S1PR1) which is required for cell egress from tissues (3) and αE integrin CD103 (which binds E-cadherin on epithelial cells). The vast majority of T_M_ in peripheral blood are CD69−/CD103– whereas in most human tissues, T_M_ express high levels of CD69. However, the expression of CD103 is mainly confined to CD8+ T_M_ in mucosal sites (4–7). New evidences have suggested that T_RM_ not only plays an important role in generating antigen specific responses to pathogens but importantly vaccine-generated T_RM_ can mediate cross-strains protection, and persist for long periods of time after vaccination (8).

*Salmonella enterica* serovar Typhi (*S*. Typhi), a human restricted pathogen, causes typhoid fever in ~26.9 million individuals yearly leading to around 223,000 deaths worldwide (9–12). *S*. Typhi actively invade the mucosal surfaces of the host upon ingestion and subsequently enters the submucosa where it interacts with intestinal lymphoid tissues before reaching the draining mesenteric lymph nodes where it spreads to numerous tissues leading to systemic illness (12). While *S*. Typhi has the potential to enter at any location along the intestine through M cells and epithelial cells (13), the human terminal ileum (TI) is the preferred intestinal active invasion site for *S*. Typhi (14). Not much is known regarding the generation of T_RM_ immune responses to *Salmonella* in the human terminal ileum. Thus, it is important to understand how oral vaccines (e.g., oral attenuated Typhoid vaccine, Ty21a) induce the generation of site-specific protective memory responses following vaccination resulting in prevention from disease. Currently, two licensed typhoid vaccines are available in the USA for use in humans, including Ty21a (12). Ty21a, a Gal E *S*. Typhi mutant strain, is usually given in four spaced doses and achieves a modest level of long-lived protection (60–80%, 5–7 years) (12, 15–17).

Numerous studies have examined extensively the induction of humoral and B and T cell-mediated immunity (CMI) responses in peripheral blood mononuclear cells (PBMC) collected from healthy individuals following Ty21a vaccination and other attenuated *S*. Typhi vaccine candidates (17–31). Recently, we have reported that oral Ty21a immunization elicits significant *S*. Typhi-specific CD8+ T_M_–(T_CM_, T_EM_, and T_EMRA_) responses with multiple functions (cytotoxic—CD107a—, IFN-γ, IL-17A, and TNF-α) which are unique in human terminal ileum (TI) lamina propria mononuclear cells (LPMC) (32). Given that a majority of the CD8+ T_M_ in the TI mucosa may express the markers for tissue resident T cells, we hypothesized that oral Ty21a immunization would modulate CD8+ T_RM_ and that most of the *S*. Typhi-specific responses observed at the terminal ileum mucosa would be mediated by CD8+ T_RM_.

CD8+ T_RM_ express abundantly the integrin αE (CD103), which binds to E-cadherin on intestinal epithelial cells (IEC). It is reasonable to hypothesize that these CD8+ T_RM_ are poised to migrate to the epithelium compartment and contribute to *S*. Typhi immunity in the TI mucosa. No information is available concerning the role of the intraepithelial (IEL) CD8+ T_RM_ following oral Ty21a vaccination, largely due to the fact that functional studies with human intestinal IEL are challenging because of the low IEL cell numbers obtained. However, since IEL are likely to contribute to the first line of defense, it is important to understand their role and contribution in oral Ty21a vaccination and *S*. Typhi infection.

In this study we have characterized CD8+ T_RM_ from lamina propria (LPMC) and the epithelium (IEL) obtained from terminal ileum biopsies of Ty21a vaccinated and unvaccinated volunteers. We evaluated and compared CD8+ T_RM_ subsets *ex-vivo* and the *S*. Typhi-specific responses in the two groups of participants following stimulation with autologous target cells infected with wt *S*. Typhi. Finally, we determined the multifunctionality of the elicited responses, i.e., their ability to concomitantly exhibit more than 1 function [e.g., interferon (IFN)-γ, tumor necrosis factor (TNF)-α, IL-2 and IL-17A]. These comparisons provide unique insights into the responses generated at the mucosal level by CD8+ T_RM_ subsets following oral Ty21a immunization.

## Materials and Methods

### Ty21a-Immunization and Sample Collection

**Figure 1:**
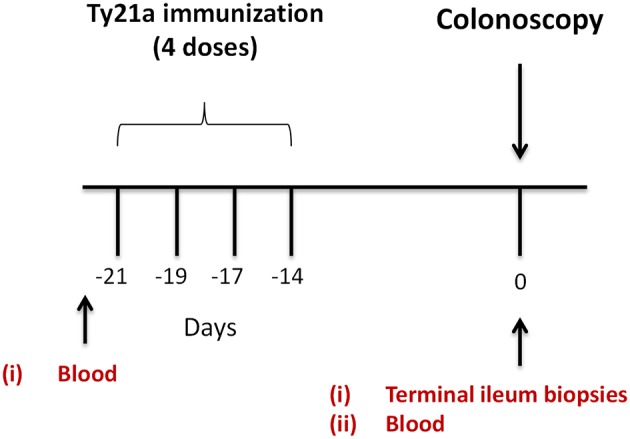
Study design. Oral typhoid vaccine Ty21a dose schedule (4 doses at −21 to −14 days) and time of collection of specimens (blood and terminal ileum (TI) biopsies) from volunteers undergoing routine screening colonoscopies. Autologous EBV-B cells were generated from pre-immunized blood.

Volunteers (Demographics shown in Table S1) were allocated into two groups. The first (*n* = 17) were given orally the Ty21a vaccine (4 doses) (Vivotif enteric-coated capsules; Crucell, Bern, Switzerland). The second group consisted of individuals who were unvaccinated (control group) (*n* = 20) as described in the study design (Figure 1). Pre-immunized peripheral blood (at least 21 days before colonoscopy) were obtained and on colonoscopy day (day 0), blood and TI biopsies using large capacity forceps were obtained (Figure 1). PBMC were isolated using density gradient centrifugation and cryopreserved using established methods (29).

### Isolation of Lamina Propria Mononuclear Cells (LPMC) and Intraepithelial Lymphocytes (IEL) From Terminal Ileum Biopsies

Terminal ileum LPMC and IEL were isolated as previously reported (33–35). Briefly, terminal ileum biopsies were collected from volunteers undergoing screening colonoscopy. Biopsies were digested with HBSS (without CaCl_2_, MgCl_2_, MgSO_4_; Gibco, Carlsbad, CA) and 10 mM EDTA (Ambion, Grand Island, NY) while being vigorously shaken for 45 min to isolate IEL. Next, the biopsies were digested enzymatically with collagenase D (100 μg/mL; Roche, Indianapolis, IN) and DNase I (10 μg/mL; Affymetrix, Cleveland, OH) for 45 min. The tissue was then homogenized using a Bullet Blender homogenizer (Next Advance Inc., Averill, NY) to extract LPMC. Subsequently, LPMC were washed and placed into complete medium(cRPMI) (RPMI 1640 [Gibco Invitrogen, Carlsbad, CA] which is composed of 10% heat-inactivated fetal bovine serum [BioWhittaker, Walkersville, MD], 2 mM l-glutamine [HyClone, Logan, UT], 2.5 mM sodium pyruvate [Gibco], 10 mM HEPES [Gibco], 100 U/mL penicillin [Sigma-Aldrich, St. Louis, MO], 100 μg/mL streptomycin [Sigma-Aldrich], and 50 μg/mL gentamicin [Gibco]). Viable number of isolated LPMC were then enumerated using Kova Glastic Slides (Hycor Biomedical, Garden Grove, CA). Finally, LPMC and IEL were stained to phenotype the cells and/or stimulated overnight.

### Generation of Autologous Target Cells

Using each volunteer's pre-vaccinated PBMC, autologous Epstein-Barr virus (EBV)-transformed lymphoblastoid cell line (EBV-B cells) were produced (Figure 1) as previously described (19, 29).

### Infection of Autologous EBV-B With *S*. Typhi

The generated autologous target cells (EBV-B) were infected with the wild type strain of *S*. Typhi ISP1820 at a MOI 7:1 for 3 h at 37°C in plain RPMI. After the infection, the cells were washed thrice with cRPMI and cultured overnight with cRPMI including 150 μg/mL of gentamicin. The cells were washed again and the efficiency of *S*. Typhi infection was determined using the anti-*Salmonella* common structural Ag (CSA-1) polyclonal antiserum conjugated to FITC (Kierkegaard and Perry, Gaithersburg, MD). Cells were then stained and analyzed by flow cytometry as reported before (19, 29).

### Stimulation of Terminal Ileum LPMC and IEL

Isolated TI-LPMC and IEL were taken as effector cells as previously described (32, 34). Briefly, LPMC and IEL, respectively, were co-cultured with (i) uninfected or (ii) *S*. Typhi–infected EBV-B (MOI of 7:1). The negative controls were LPMC and IEL cultured with media only. While the positive controls involved cultures of LPMC and IEL in the presence of α-CD3/CD28 (Life technologies, Grand Island, NY). After 2 h of incubation, Golgi Stop (0.5 μl; Monensin, BD) and Golgi Plug (0.5 μl, Brefeldin A, BD) were added and the cultures continued overnight at 37°C in 5% CO_2_.

### Ty21 Homogenate

Ty21a bacteria strain was grown overnight in LB supplemented with galactose as described previously (36). The bacteria was then homogenized using a French press, and the homogenate centrifuged at 17,700 g for 10 min. The pellet was discarded and the supernatant filtered through a 0.8 μm filter and kept −20°C. Protein concentration measured with a BCA protein kit (Fisher). LPMC cells were stimulated with 10 μg/mL of Ty21a homogenate. After 2 h of incubation, Golgi Stop (0.5 μl; Monensin, BD) and Golgi Plug (0.5 μl, Brefeldin A, BD) were added and cultures continued overnight at 37°C in 5% CO_2_.

### Surface and Intracellular Staining

After overnight stimulation, TI-LPMC and IEL were stained for flow cytometry analysis as reported before (32, 33). Briefly, LPMC and IEL were stained using a viability dye (live/dead fixable yellow stain-YEVID) (Invitrogen, Carlsbad, CA) to exclude dead cells. Subsequently, Fc receptors on the cell surface were blocked using human immunoglobulin (3 μg/mL; Sigma). Surface staining was subsequently performed. LPMC and IEL were stained with fluorescently labeled monoclonal antibodies (mAbs) against CD13-Pacific Orange (conjugated in-house), CD19-BV570 (HIB19, Biolegend, San Diego, CA), CD3-BV650 (OKT3, Biolegend), CD4-PE-Cy5 (RPA-T4, BD), CD8-PerCP-Cy5.5 (SK1, BD), CD45RA-biotin (HI100, BD), CD62L-APC-A780 (DREG-56, eBioscience, San Diego, CA), and CD103-FITC (Ber-ACT8, BD) and incubated at 4°C for 30 min. This was followed by washing cells with wash buffer and staining using streptavidin (SAV)-Qdot800 (Invitrogen) for 30 min at 4°C. Afterwards, cells were fixed with IC fixation buffer for 20 min at room temperature (catalog No 8222, eBioscience). This was followed by the permeabilization of the cells using IC permeabilization buffer (catalof No 8333, eBioscience) as reported before. For intracellular staining, mAbs directed to interleukin (IL)-17A-BV421 (BL168, Biolegend), IFN-γ-PE-Cy7 (B27, BD), TNF-α-Alexa 700 (MAb11, BD), CD69-ECD (TP1.55.3, Beckman Coulter, Danvers, MA), and interleukin (IL)2-BV605 (MQ1-17H12, Biolegend) were used to stain LPMC and IEL at 4°C overnight. Stained cells were kept in 1% paraformaldehyde at 4°C until data collection using a customized LSRII flow cytometer (BD). Data were analyzed using WinList version 7 (Verity Software House, Topsham, ME). Antigen specific responses were expressed as the net percentages of positive cells calculated by subtracting the values (percentages) obtained following stimulation with *S*. Typhi-infected targets from the background values obtained after stimulation with uninfected cells. We deemed that a response is specific if the number of positive events between experimental (*S*. Typhi–infected targets) and negative control (uninfected targets) cultures was significantly higher (*p* < 0.01) by *z*-tests. MF *S*. Typhi–specific by TI LPMC and IEL following immunization were evaluated using the FCOM function of the WinList analysis package.

### Statistical Analysis

The statistical software GraphPad Prism™ version 5.03 (Graphpad, San Diego, CA, USA) was used to analyze the data. Mann–Whitney tests were used to assess statistical differences in median values between two groups. Statistical differences between LPMC and IEL paired responses were assessed by Wilcoxon matched pair tests. Spearman correlation tests were used to examine the correlations between LPMC and IEL *S*. Typhi–specific responses.

## Results

### Oral Ty21a-Immunization Does Not Alter CD8^+^-T_RM_ Frequencies

**Figure 2:**
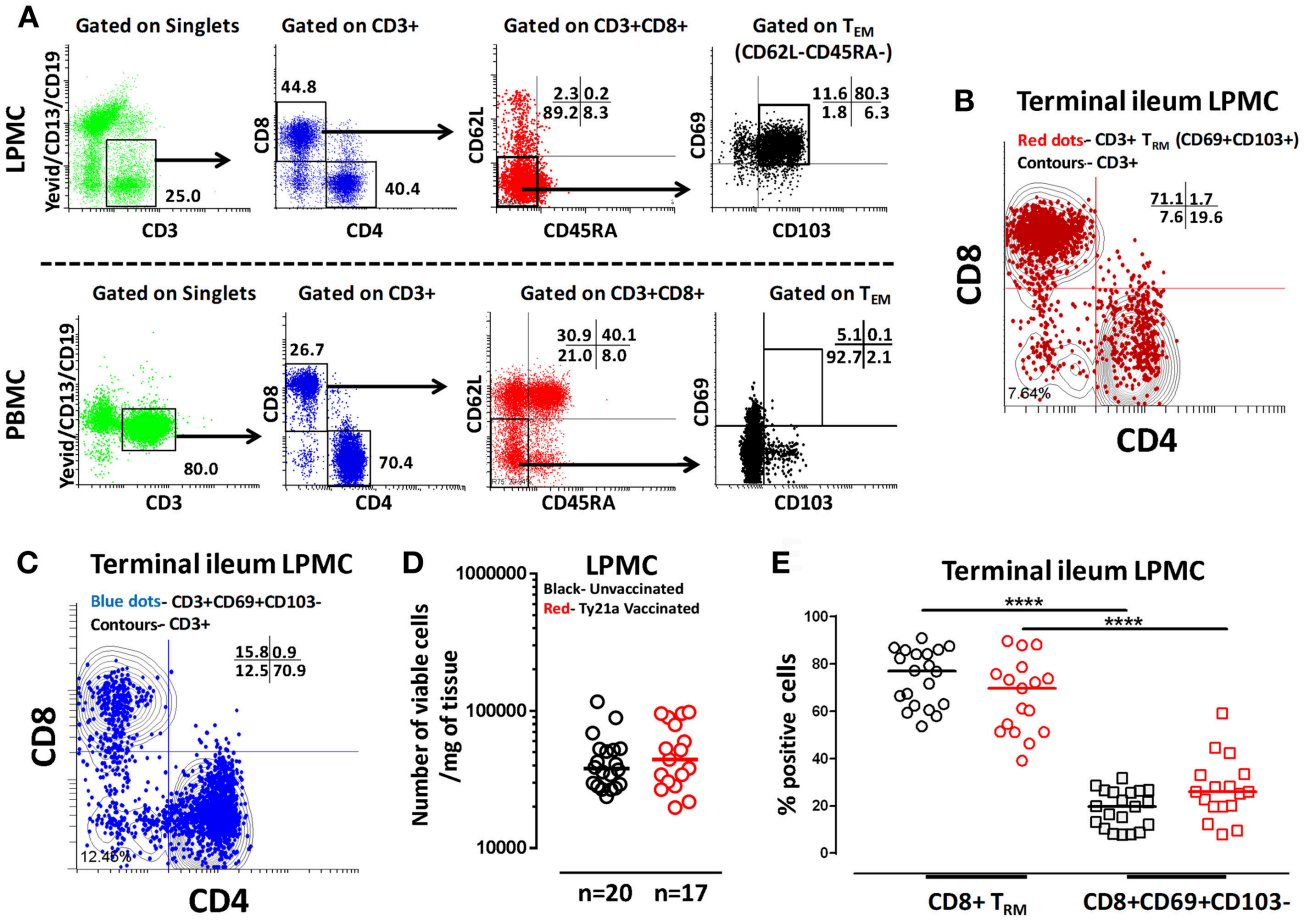
Gating Strategy and cell subset frequencies of terminal ileum tissue-resident memory T (T_RM_) cells. CD8+ tissue resident T memory cell (T_RM_) subsets from a representative Ty21a vaccinated volunteer were detected in **(A)** terminal ileum LPMC, and PBMC using CD69 and CD103 markers following the strategy shown in the figure. Cytograms from a representative Ty21a vaccinated volunteer depicting the distribution of **(B)** T_RM_ (CD3+CD69+CD103+; red dots); **(C)** CD3+CD69+CD103- (blue dots) in terminal ileum LPMC CD4+ and CD8+ subsets (contours) gated on LPMC CD3+ T cells. **(D)** The absolute number of viable LPMC per mg of tissues obtained from terminal ileum biopsies of Ty21a-vaccinated (*n* = 17; red symbols) and unvaccinated (*n* = 20; black symbols) volunteers were compared. **(E)** Frequencies of CD8+ T_RM_ and CD8+CD69+CD103– were measured and compared between TI LPMC obtained from Ty21a-vaccinated (*n* = 17; red symbols) and unvaccinated volunteers (*n* = 20; black symbols) with significant differences (****P < 0.00005) indicated. Horizontal bars represent median values.

The effects of oral Ty21a-immunization on human TI LPMC CD8+ T_RM_ function is unknown. To explore whether Ty21a-immunization influences the frequencies of CD8+ T_RM_ subsets, we first isolated TI LPMC from biopsies obtained from volunteers who either received four doses of Ty21a (*n* = 17) or were unvaccinated (*n* = 20). We then characterized CD8+ T_RM_ in TI LPMC by using CD69 and CD103 markers following the gating strategy depicted in Figure 2A. We observed that TI LPMC CD8+ T_RM_ exhibit a high frequency (~80%) of CD69+ CD103+ (T_RM_) cells while CD69+CD103– T cells constitute a minor population (~11%) in this representative volunteer (Figure 2A). As expected, circulating PBMC CD8+ T_EM_ are mostly CD69^−^ CD103^−^ (Figure 2A). The distribution of CD3+ CD69+ CD103+ (T_RM_) (shown by red dots) is confined mainly to the CD8+ T compartment with minor populations in CD4+ and CD4– CD8– (Figure 2B), while CD3+CD69+CD103– (shown by blue dots) is observed mostly in the CD4+ T subset (Figure 2C). In this study, we will focus on the observations in CD8+ T_RM_ and CD8+CD69+CD103– T cells subsets. The responses of the CD4+ T_RM_ subsets have been compiled in a separate manuscript currently under review. To determine whether the LPMC cell yields obtained from biopsies from Ty21a-vaccinated and unvaccinated volunteers were similar, we compared the number of viable cells per mg of tissue. No differences were observed in either the numbers of LPMC or CD8+ T_RM_ between the two groups (Figures 2D,E). As expected, the cumulative frequencies of CD8+CD69+CD103– T cells were significantly lower than CD8+ T_RM_ in both groups of participants (Figure 2E).

### Activation of Terminal Ileum LPMC CD8+ T_RM_ and CD8+CD69+CD103– T Cells

Most of our knowledge of CMI responses elicited by *S*. Typhi infection or Ty21a-immunization in humans is based almost exclusively on data derived from blood (17, 25). We have recently reported the first study on LPMC CD8+ T_M_ (T_EM_, T_CM_, and T_EMRA_) immune responses following Ty21a oral immunization (32). However, the role and contribution of TI CD8+ T_RM_ remains unknown following oral Ty21a immunization or wt *S*. Typhi infection.

**Figure 3:**
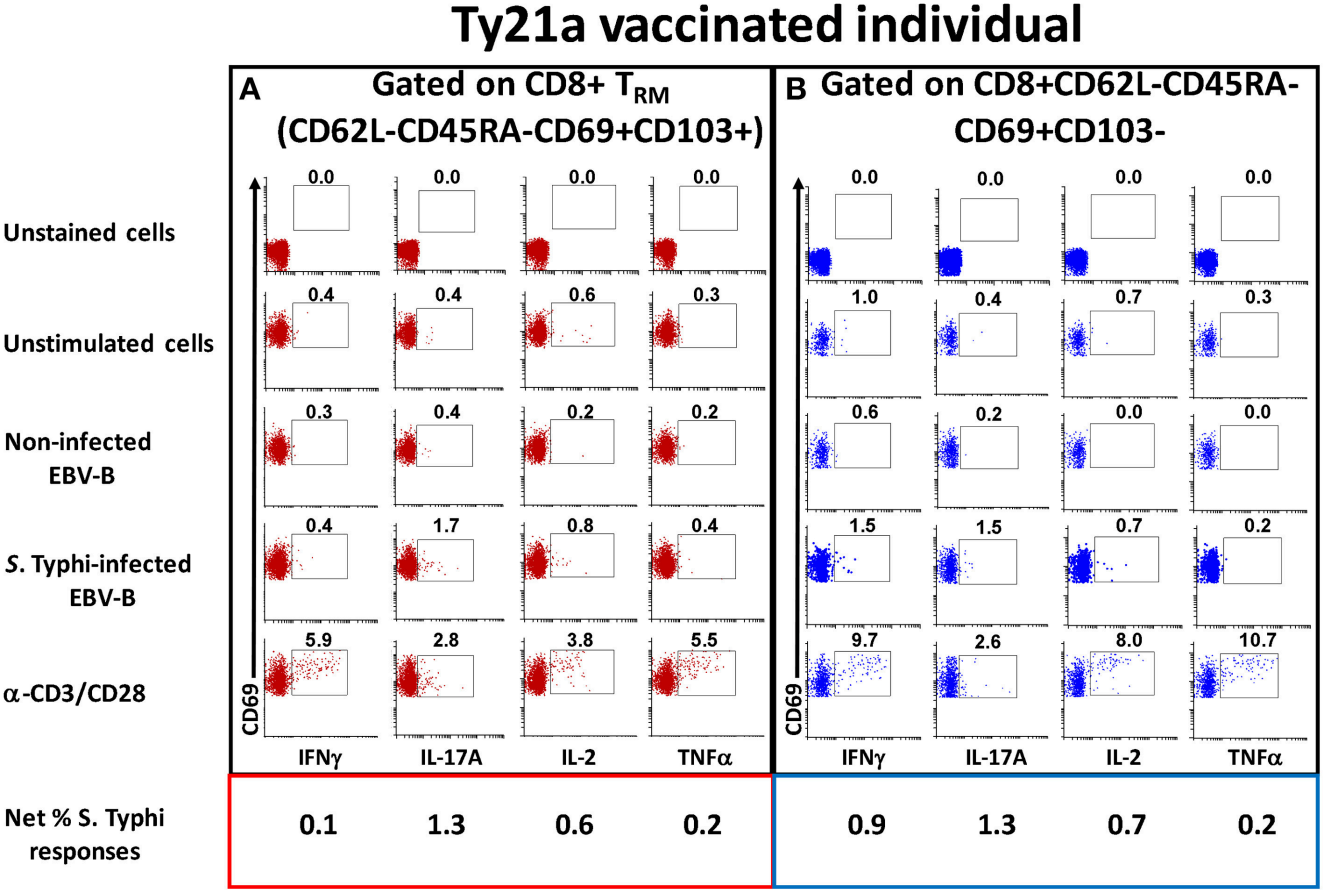
Spontaneous and *S*. Typhi-specific responses in LPMC CD8+ T cell subsets isolated from terminal ileum of a Ty21a-vaccinated representative volunteer. **(A)** CD8+ T_RM_ and **(B)** CD8+CD69+CD103–T cells were stimulated with non-infected or *S*. Typhi-infected autologous EBV-B cells and produced cytokines (IFN-γ, IL-17A, IL-2, and TNF–α). Anti (α)-CD3/CD28 stimulation was used as a positive control in both subsets while unstimulated LPMC CD8+ T_RM_ or CD8+CD69+CD103–T cells alone were used as negative controls. In addition, unstained cell controls were used to place the delimiters for LPMC auto-fluorescence. The percentage of positive cells in the gated regions is shown above the corresponding black boxes.

To address this important gap, we determined the ability of LPMC CD8+ T_RM_ and CD8+CD69+CD103– T cells obtained from Ty21a vaccinated (*n* = 17) and unvaccinated (*n* = 20) volunteers to be activated following co-culture with autologous *S*. Typhi-infected or uninfected EBV-B cells or media alone or α-CD3/CD28 by assessing their cytokines responses following overnight stimulation. Cytograms in Figure 3 depict LPMC CD8+ T cell subset responses from a representative Ty21a vaccinated individual (Figure 3A CD8+ T_RM_ (CD69+CD103+) and Figure 3B CD8+CD69+CD103–). Interestingly, we observed the presence of baseline levels of cytokine production (IFN-γ, IL-17A, IL-2, and TNF-α) in unstimulated LPMC CD8+ T_RM_ and CD8+CD69+CD103– subsets (Figures 3A,B). Following stimulation with *S*. Typhi-infected targets, we observed substantial net increases (% of *S*. Typhi-infected EBV-B responses—% of uninfected EBV-B responses) in the frequencies of CD8+ T_RM_ and CD8+CD69+CD103– T cells (e.g., IFN-γ, IL-17A, IL-2, TNF-α) (Figures 3A,B). Interestingly, the levels of individual cytokines varied depending on the LPMC subset evaluated. Cytograms in Figure S1 depict LPMC CD8+ T cell subset responses from a representative unvaccinated individual (Figures S1A CD8+ T_RM_ (CD69+CD103+) and Figures S1B CD8+CD69+CD103–). Remarkably, we also observed the presence of baseline levels of cytokine (IFN-γ, IL-17A, IL-2, and TNF-α) in unstimulated LPMC CD8+ T_RM_ and CD8+CD69+CD103– subsets (Figures S1A,B). However, following stimulation with *S*. Typhi-infected targets, we observed no net increases in the frequencies of CD8+ T_RM_ and CD8+CD69+CD103– T cells cytokine (IFN-γ, IL-17A, IL-2, and TNF-α) producing cells (Figures S1A,B).

### Oral Ty21a Immunization Induces Terminal Ileum LPMC CD8+ T_RM_ and CD8+CD69+CD103– T Cells at Baseline

**Figure 4:**
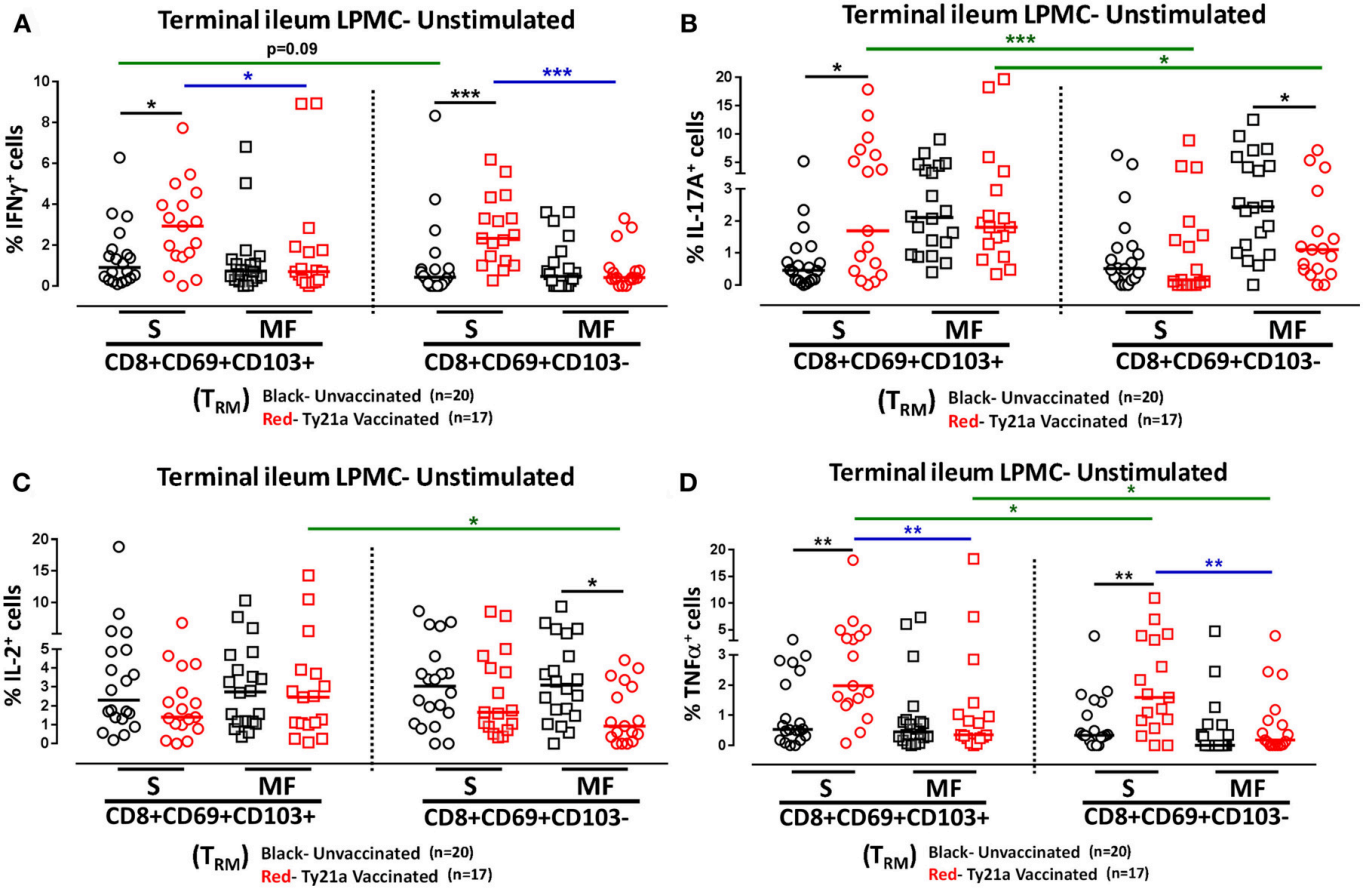
Effect of oral Ty21a-immunization on terminal ileum LPMC CD8+ T_RM_ and CD8+CD69+CD103–T cells spontaneously expressing one or more cytokines. *Ex-vivo* unstimulated CD8+ T_RM_ and CD8+CD69+CD103–T cells were cultured overnight and their cytokine (IFN-γ, IL-17A, IL-2, and TNF-α) production were determined by flow cytometry. Using the FCOM function of Winlist, CD8+ T_RM_ and CD8+CD69+CD103–T responses were stratified into multifunctional cells (MF) and single-positive cells (S). Comparison of TI LPMC CD8+ T_RM_ and CD8+CD69+CD103–T responses in **(A)** INF-γ+; **(B)** IL-17A+; **(C)** IL-2+, and **(D)** TNF-α+ MF and S in Ty21a-vaccinated (*n* = 17; red symbols) and unvaccinated volunteers (*n* = 20; black symbols) with significant differences shown (*P < 0.05; **P < 0.005; ***P < 0.0005). Black lines: significant differences between Ty21a vaccinated and unvaccinated volunteers. Blue lines: significant differences between S and MF. Green lines: significant differences between CD8+ T_RM_ and CD8+CD69+CD103– T cell responses. Horizontal bars (black and red) represent median values.

We next investigated whether oral Ty21a immunization non-specifically activates CD8+ T_RM_ and CD8+CD69+CD103– T cells. To this end we used the FCOM function of WinList to determine and compared the frequency of single (**S**) and multifunctional (MF) producing cytokines (IFN-γ, IL-17A, IL-2, and TNF-α) in CD8+ T_RM_ and CD8+CD69+CD103– T after freshly isolated LPMC were cultured unstimulated overnight. We found that *ex-vivo* unstimulated LPMC CD8+ T_RM_ and CD8+CD69+CD103– T cells obtained from Ty21a immunized volunteers exhibited significantly higher level of single-producing IFN-γ cells than unimmunized volunteers (Figure 4A). No differences in the frequency of unstimulated LPMC CD8+ T_RM_ and CD8+CD69+CD103– T MF IFN-γ were noted for either group (Figure 4A). Next, we observed that *ex-vivo* unstimulated LPMC CD8+ T_RM_ obtained from Ty21a immunized volunteers exhibited significant increases in IL-17A-S but not IL-17A-MF (Figure 4B). In contrast, *ex-vivo* unstimulated LPMC CD8+CD69+CD103– T cells showed decreases in IL-17A responses following Ty21a immunization with significantly (*p* < 0.05) lower levels in IL-17AMF (Figure 4B). Subsequent comparisons of the levels of IL-17A S and MF production LPMC CD8+ T_RM_ and CD8+CD69+CD103– T cells revealed significantly (*p* < 0.05) higher IL-17A S and MF production in LPMC CD8+ T_RM_ following Ty21a immunization (Figure 4B). We then determined IL-2 in unstimulated LPMC and found that there were no differences in the frequency of IL-2+ S and MF in LPMC CD8+ T_RM_ following Ty21a vaccination (Figure 4C). In contrast, CD8+CD69+CD103– T cells produce significantly lower IL-2 MF following Ty21a vaccination (Figure 4C). Additionally, we observed significantly (*p* < 0.05) higher level of IL-2 MF in LPMC CD8+ T_RM_ than in CD8+CD69+CD103– T cells (Figure 4C). Finally, we observed that *ex-vivo* unstimulated LPMC CD8+ T_RM_ and CD8+CD69+CD103– T obtained from Ty21a immunized volunteers have significantly higher levels of TNF-α S than unimmunized volunteers (Figure 4D). However, TNF-α S and MF levels were significantly higher in CD8+ T_RM_ than in CD8+CD69+CD103– T cells following Ty21a vaccination (Figure 4D).

Because CD8+ T_RM_ and CD8+CD69+CD103–T cells showed differences in cytokine production without *ex vivo* stimulation following Ty21a immunization, we hypothesized that LPMC CD8+ T_RM_ and CD8+CD69+CD103–T cells isolated from Ty21a-vaccinated and unvaccinated volunteers may have intrinsic changes in their activation capacity. Therefore, we assessed whether α-CD3/CD28 beads would stimulate LPMC CD8+ T_RM_ and CD8+CD69+CD103–T cells equally. To this end we used the Winlist FCOM function to determine and compare the frequencies of single (S) and multifunctional (MF) producing cytokines (IFN-γ, IL-17A, IL-2, and TNF-α) in CD8+ T_RM_ and CD8+CD69+CD103– T cells in freshly isolated LPMC stimulated with α-CD3/CD28 beads overnight. We found no differences in IFN-γ+ S and MF frequencies between Ty21a immunized and unimmunized volunteers in LPMC CD8+ T_RM_ and CD8+CD69+CD103– T cells following α-CD3/CD28 stimulation (Figure S2A). In contrast, after α-CD3/CD28 stimulation, we observed that LPMC CD8+ T_RM_ obtained from Ty21a immunized volunteers have significant increases in IL-17A S but not IL-17A MF when compared to unimmunized volunteers (Figure S2B). Remarkably, after α-CD3/CD28 stimulation, the frequency of LPMC CD8+CD69+CD103– IL-17A+ MF decreases significantly following Ty21a immunization (Figure S2B). Further, we observed significantly (*p* < 0.05) higher levels of IL-17A S and MF in LPMC CD8+ T_RM_ than in CD8+CD69+CD103– T cells after α-CD3/CD28 stimulation (Figure S2B). Regarding IL-2 production, no differences in IL-2+ S and MF frequencies were found between Ty21a immunized than unimmunized volunteers in LPMC CD8+ T_RM_ (Figure S2C). In contrast, after α-CD3/CD28 stimulation, the frequency of LPMC CD8+CD69+CD103– IL-2+ MF decreases significantly following Ty21a immunization (Figure S2C). Finally, we measured TNF-α production and found no differences in TNF-α+ S and MF frequencies between Ty21a immunized than unimmunized volunteers in LPMC CD8+ T_RM_ (Figure S2D). However, after α-CD3/CD28 stimulation, the frequency of LPMC CD8+CD69+CD103– IL-2+ S increases significantly following Ty21a immunization (Figure S2D). These results indicate that oral Ty21a immunization may modulate LPMC CD8+ T_RM_ (IL-17A) and CD8+CD69+CD103–T cells (IL-17A, IL-2 and TNF-α) to exhibit intrinsic differences in their ability to respond to α-CD3/CD28 stimulation.

### Oral Ty21a-Immunization Elicits Differential Terminal Ileum LPMC *S*. Typhi Responsive CD8+ T_RM_ and CD8+CD69+CD103– T Cells

**Figure 5:**
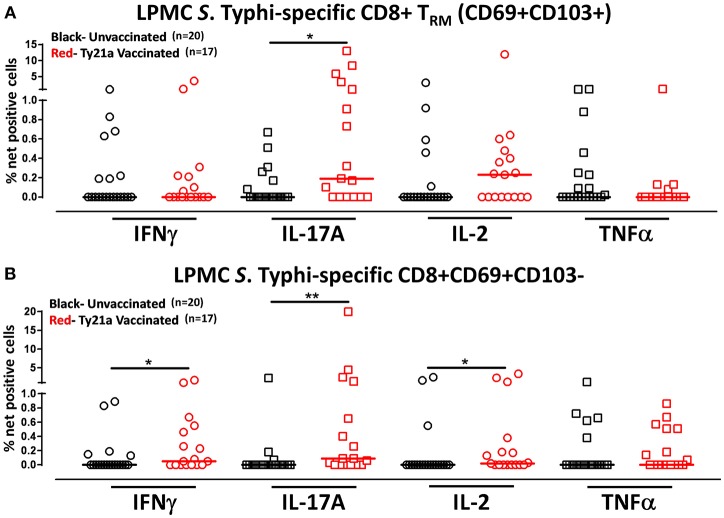
*S*. Typhi-specific responses of terminal ileum LPMC CD8+ T_RM_ and CD8+CD69+CD103– T cell subsets in healthy adults following oral Ty21a-immunization. The net percentages of *S*. Typhi-specific responses (IFN-γ, IL-17A, IL-2, and TNF-α) in **(A)** CD8+ T_RM_ and **(B)** CD8+CD69+CD103– T cell subsets were compared between Ty21a-vaccinated (*n* = 17; red symbols) and unvaccinated volunteers (*n* = 20; black symbols) with significant differences (*P < 0.05; **P < 0.005) indicated. Horizontal bars (black and red) represent median values.

Although CD8+ T_RM_ is one of the major subsets located at the site of infection (terminal ileum for *S*. Typhi), little is known about its role and contribution in the locally elicited *S*. Typhi-specific responses following oral Ty21a immunization. We hypothesized that CD8+ T_RM_ would respond strongly and contribute to most of the elicited responses while CD8+CD69+CD103–T cells would also respond albeit differently in magnitude and characteristics following Ty21a immunization. To test this hypothesis, we evaluated the ability of CD8+ T_RM_ and CD8+CD69+CD103–T cells obtained from TI biopsies of Ty21-vaccinated and unvaccinated volunteers to elicit *S*. Typhi-specific responses following stimulation with autologous *S*. Typhi-infected and uninfected targets cells. Cumulative data of *S*. Typhi-specific CD8+ T_RM_ responses expressed as net percentage of positive cells are shown in Figure 5A. Following Ty21a vaccination, TI LPMC CD8+ T_RM_ exhibited significantly (*p* < 0.05) higher *S*. Typhi-specific IL-17A responses than CD8+ T_RM_ obtained from unvaccinated volunteers (Figure 5A). This is, to our knowledge, the first demonstration of local *S*. Typhi-specific CD8+ T_RM_ responses in the TI and suggesting that CD8+ T_RM_ are primarily T_C_17 in the local mucosa following oral Ty21a-immunization.

Because CD8^+^ T_RM_ appears to be mostly IL-17A effectors, we also evaluated the role and contribution of CD8+CD69+CD103– T cells to the local responses following Ty21a immunization and *ex vivo* stimulation with *S*. Typhi-infected targets. Cumulative data of *S*. Typhi-specific CD8+CD69+CD103– T responses expressed as net percentage of positive cells are shown in Figure 5B. Interestingly, we observed that TI LPMC CD8+CD69+CD103–T cells obtained from Ty21a-vaccinated volunteers exhibited significantly (*p* < 0.05) higher *S*. Typhi-specific cytokine production (IFN-γ, IL-17A, and IL-2) responses than CD8+CD69+CD103–T cells obtained from unvaccinated volunteers (Figure 5B). We conclude that LPMC CD8+CD69+CD103–T cells also contribute to the *S*. Typhi-specific elicited following Ty21a vaccination.

### Multifunctional Terminal Ileum LPMC CD8+ T_RM_ and CD8+CD69+CD103–T Cells Responses Following Oral Ty21a-Immunization

**Figure 6:**
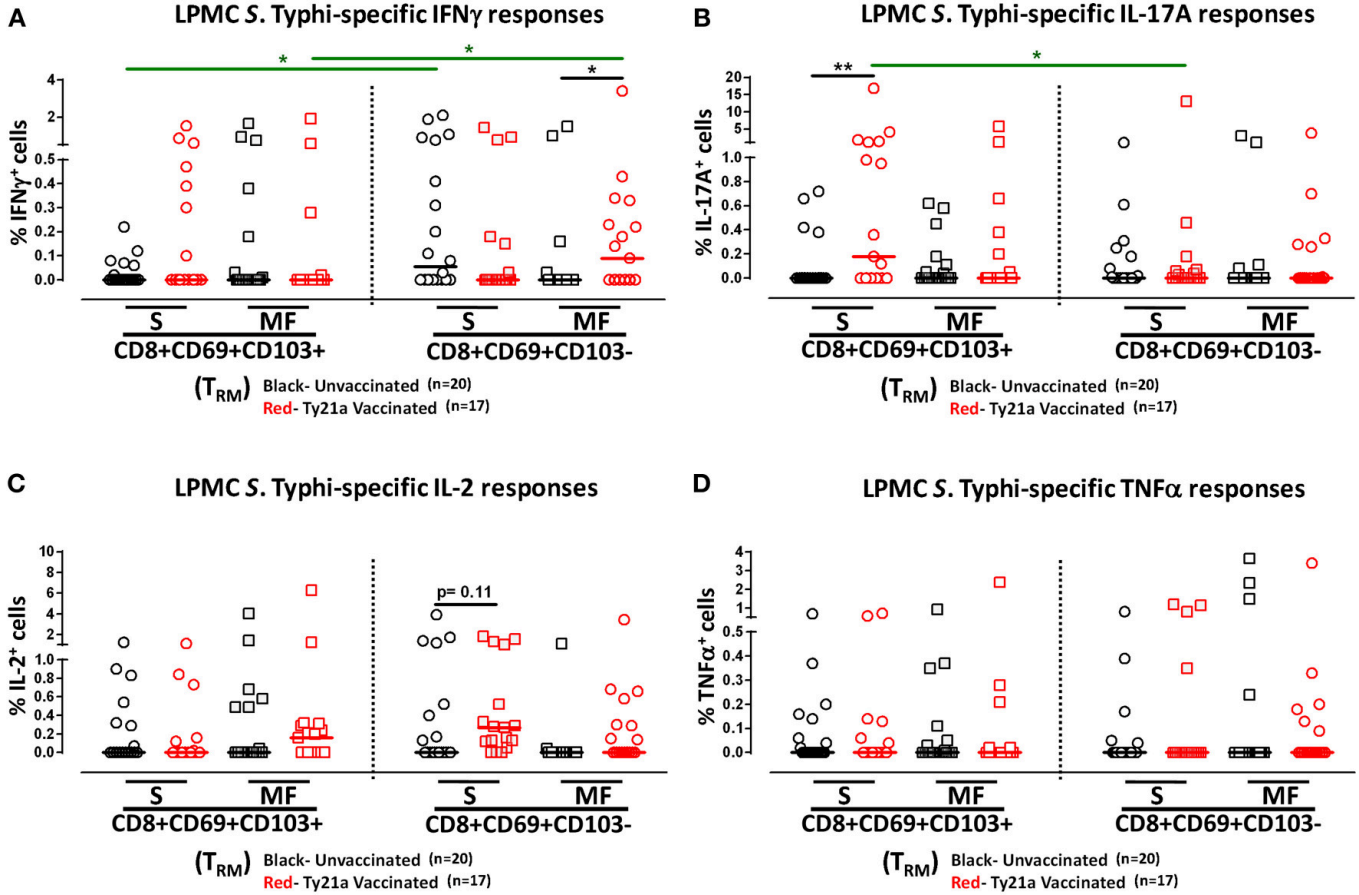
Multifunctional and single cytokine-expressing *S*. Typhi-specific CD8+ T_RM_ and CD8+CD69+CD103– T cell subsets in the terminal ileum of Ty21a-vaccinated and unvaccinated volunteers. Net *S*. Typhi-specific CD8+ T_RM_ responses were calculated using the FCOM function of Winlist and stratified into multifunctional cells (MF) and single-positive cells (S). Comparison of TI LPMC CD8+ T_RM_ and CD8+CD69+CD103– *S*. Typhi-specific **(A)** INF-γ+; **(B)** IL-17A+; **(C)** IL-2+, and **(D)** TNF-α+ MF and S in Ty21a-vaccinated (*n* = 17; red symbols) and unvaccinated volunteers (*n* = 20; black symbols). Significant differences are shown (*P < 0.05, **P < 0.005). Black lines: significant differences between Ty21a vaccinated and unvaccinated volunteers. Green lines: significant differences between CD8+ T_RM_ and CD8+CD69+CD103– T cell responses. Horizontal bars (black and red) represent median values.

**Figure 7:**
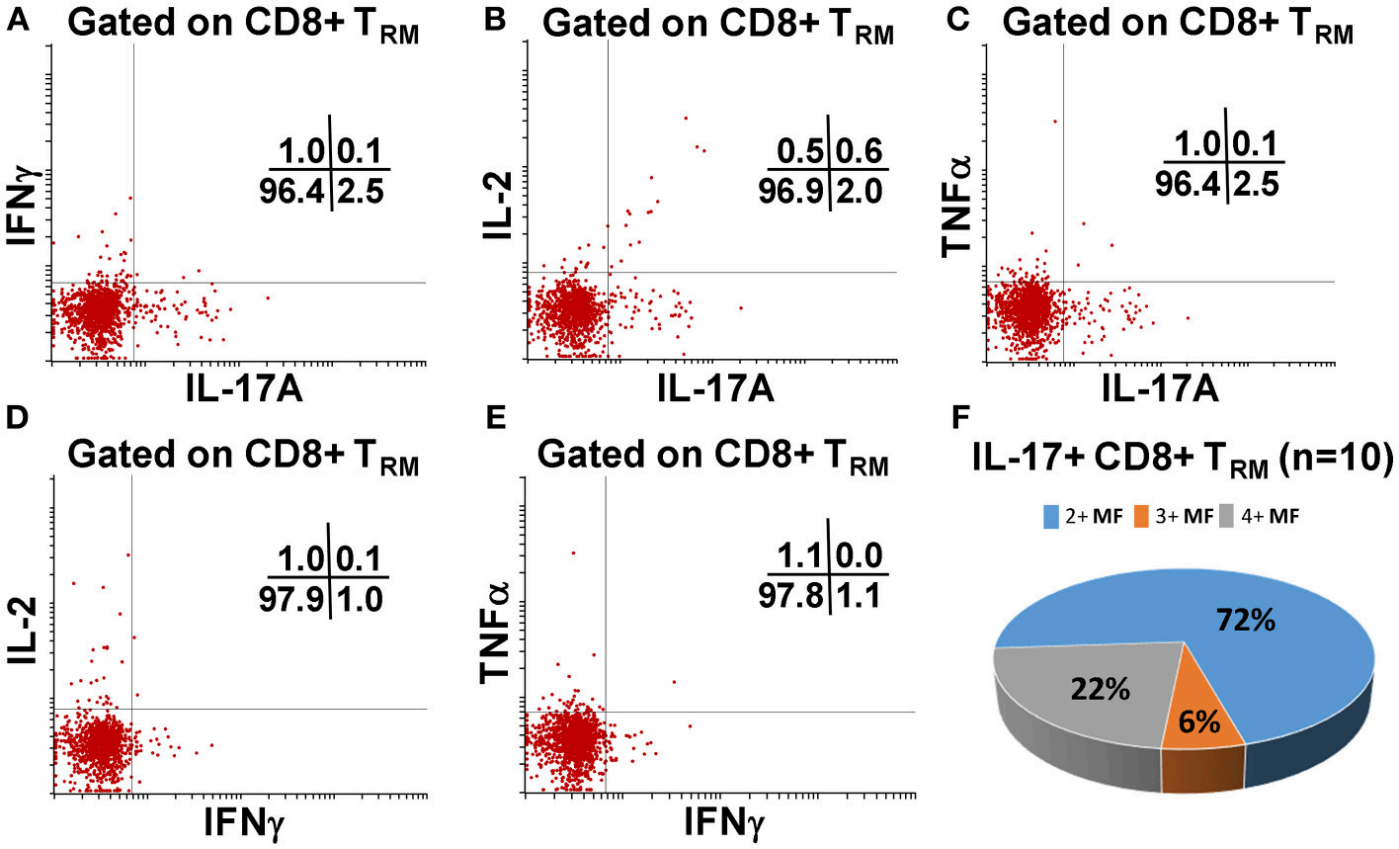
Terminal ileum *S*. Typhi-specific multifunctional CD8+ IL-17A+ T_RM_ profiles following Ty21a immunization. Cytograms from a Ty21a representative volunteer showing terminal ileum CD8+ T_RM_ response profiles for **(A)** IL-17A+ and IFN-γ+; **(B)** IL-17A+ and IL-2+; **(C)** IL-17A+ and TNF-α+; **(D)** IFN-γ+ and IL-2+; and **(E)** IFN-γ+ and TNF-α+ following stimulation with *S*. Typhi-infected targets. **(F)** Multifunctional CD8+ IL-17+ T_RM_ were stratified based on the expression of two (2+ MF), three (3+ MF), or four (4+ MF) effector functions (i.e., co-production of IL-17A with IFN-γ, IL-2, and/or TNF-α). The percentages of each subset were calculated as the proportion of the observed increases in net *S*. Typhi-specific IL-17A+ MF cells by combining cells expressing 2+, 3+, and 4+ functions in each volunteer. Data shown are the mean of *S*. Typhi-specific CD8+ IL-17A+ MF T_RM_ in responders to Ty21a vaccination (*n* = 10).

We have previously shown that PBMC and TI LPMC CD8+ T_EM_ respond to *S*. Typhi by secreting multiple cytokines simultaneously (17, 32, 37, 38). However, it is unknown whether TI *S*. Typhi-specific CD8+ T_RM_ cells responses also exhibit multi-functionality. This information will be important in uncovering the granularity of the *S*. Typhi responses in the local mucosa. Thus, we next investigated the multi-functionality of LPMC *S*. Typhi-specific CD8+ T_RM_ and CD8+CD69+CD103–T responses in Ty21a-vaccinated and unvaccinated volunteers. Using the Winlist FCOM function, TI-LPMC CD8+ T_RM_ and CD8+CD69+CD103–T responses were analyzed for multiple cytokines/chemokines (IFN-γ, IL-17A, IL-2, and TNF-α (16 possible combinations) and characterized *S*. Typhi-specific responding cells as either single cytokine producers (S) or multifunctional (Sum of double, triple, quadruple, cytokine producers) (MF). First, we analyzed LPMC *S*. Typhi-specific CD8+ T_RM_ responses associated with IFN-γ production and observed that CD8+ T_RM_ IFN-γ+ S or MF responses showed no significant differences following Ty21a vaccination (Figure 6A). In contrast, CD8+CD69+CD103– IFN-γ+ responses were prominently MF following Ty21a vaccination (Figure 6A). Furthermore, the frequency of CD8+CD69+CD103– IFN-γ+ MF from Ty21a vaccinees was significantly higher (*P* < 0.05) than their unvaccinated counterparts (Figure 6A). The level of IFN-γ+ MF was significantly higher (*P* < 0.05) in CD8+CD69+CD103– T cells than in CD8+ T_RM_ (Figure 6A). Remarkably, we observed significant increases in LPMC *S*. Typhi-specific CD8+ T_RM_ IL-17A+ S but not MF following Ty21a vaccination (Figure 6B). In contrast, no significant changes in the levels of *S*. Typhi-specific CD8+CD69+CD103– IL-17A+ S and MF was noted (Figure 6B). Moreover, we found that *S*. Typhi-specific IL-17A+ S and MF production was significantly higher (*P* < 0.05) in CD8+ T_RM_ than in CD8+CD69+CD103–T cells (Figure 6B). Likewise, comparisons were made for IL-2 (Figure 6C) and TNF-α (Figure 6D) on both cell types. No differences were noted in *S*. Typhi-specific responses in IL-2+ S and MF (Figure 6C), and TNF-α+ S and MF cells (Figure 6D) except for a higher trend in CD8+CD69+CD103– IL-2+ S responses (Figure 6C) following Ty21a immunization. We next investigated the multifunctional nature of CD8+ T_RM_ responses, particularly related to IL-17A. In Figures 7A–E we show two-parameter cytograms from a representative Ty21a volunteer. We observed that IL-17A+ CD8+ T_RM_ was co-produced mostly with IL-2 (0.6%), as compared to concomitantly with IFN-γ (0.1%) or TNF-α (0.1%) (Figures 7A–C). Low levels of cytokine co-producing CD8+ T_RM_ were observed for IFN-γ+ IL-2+ (0.1%) (Figure 7D) and IFN-γ+ TNF-α+ (0.0%) (Figure 7E). Finally, we stratified and compared the multifunctional IL-17A+ CD8+ T_RM_ responses based on the concomitant expression of two (2+ MF), three (3+ MF) or four (4+ MF) effector functions (i.e., co-production of IL-17A with IFN-γ, IL-2 and/or TNF-α). Data are shown as the mean of *S*. Typhi-specific CD8+ IL-17A+ MF T_RM_ in the volunteers who responded to Ty21a vaccination (*n* = 10) (Figure 7F). Interestingly, we observed that the majority of the MF responses were of the double-producing (72%) MF subsets (e.g., IL-17A+ IL-2+, IL-17A+ IFN-γ+ or IL-17A+ TNF-α+) (Figure 7F). The quadruple MF subsets represented about a quarter (22%) of the MF responses while the triple MF subsets represented only 6% of the MF responses (Figure 7F). We concluded that the CD8+ IL-17A+ MF T_RM_ responses were primarily double-producing cells.

### Terminal Ileum LPMC CD8+ T_RM_ and CD8+CD69+CD103– T Cells Induced *S*. Typhi-Specific Responses Following Stimulation With Soluble Ty21a Homogenate Antigens

**Figure 8:**
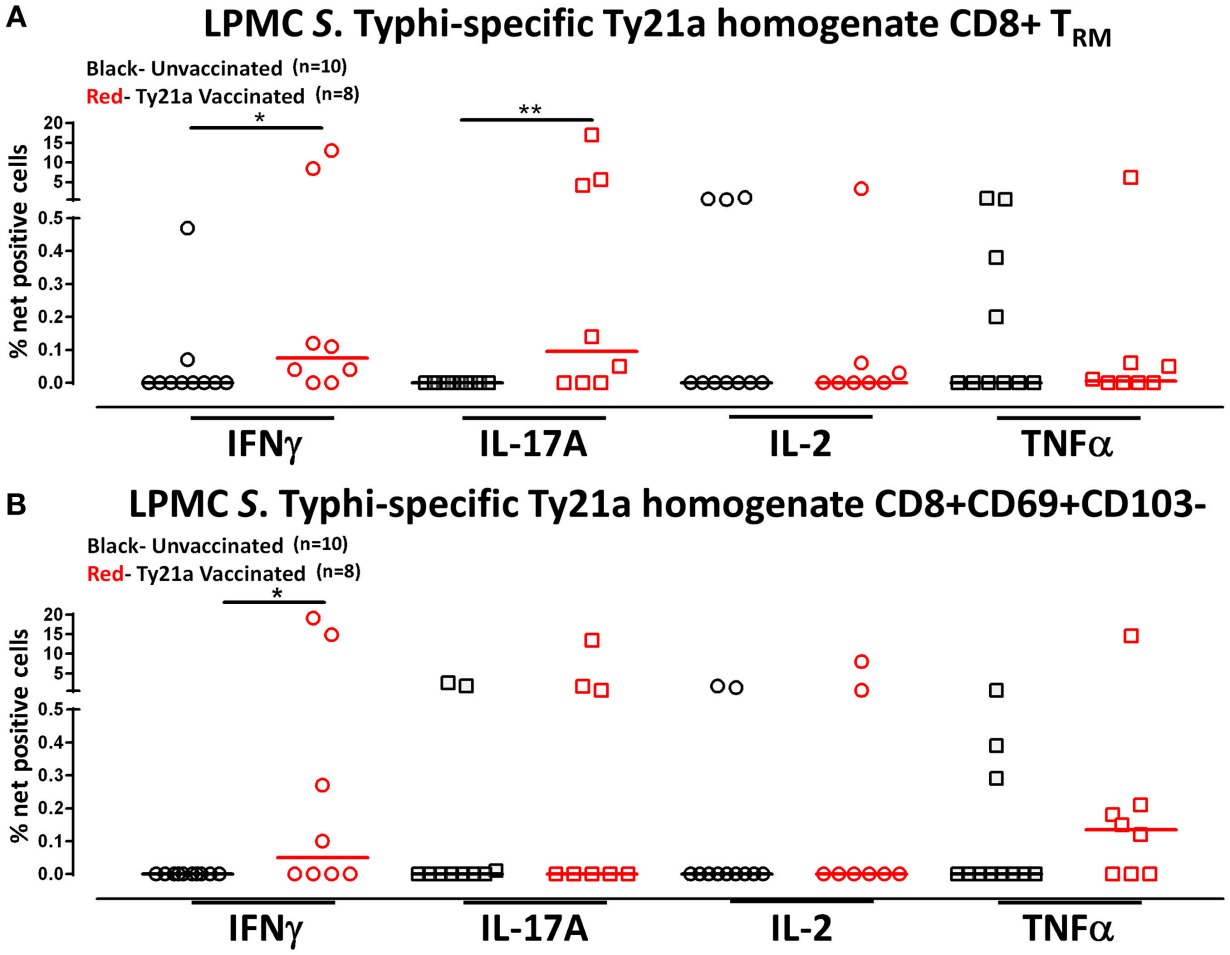
*S*. Typhi-specific responses to a Ty21a homogenate by terminal ileum LPMC CD8+ T_RM_ and CD8+CD69+CD103– T cell subsets in healthy adults following oral Ty21a-immunization. Terminal ileum LPMC CD8+ T_RM_ and CD8+CD69+CD103– T cells were stimulated with a Ty21a homogenate (10 μg/mL). The net percentages of Ty21a homogenate (with media subtracted) *S*. Typhi-specific responses (IFN-γ, IL-17A, IL-2, and TNF-α) in **(A)** CD8+ T_RM_ and **(B)** CD8+CD69+CD103– T cell subsets were compared between Ty21a-vaccinated (*n* = 8; red symbols) and unvaccinated volunteers (*n* = 10; black symbols) with significant differences shown (*P < 0.05; **P < 0.005). Horizontal bars (black and red) represent median values.

Human T_RM_ have been shown to have the capacity to respond rapidly to activation at the site of infection (39). Thus, we hypothesized that CD8+ T_RM_ and CD8+CD69+CD103– T cells may also have the ability to respond to soluble Ty21a antigens, albeit differently, as compared to *S*. Typhi-infected target stimulation. To test this hypothesis, we evaluated the ability of CD8+ T_RM_ and CD8+CD69+CD103– T cells to elicit responses following stimulation with 10 μg/ml of Ty21a homogenate antigens and using unstimulated cells as control in a subset of Ty21a vaccinated (*n* = 8) and unvaccinated (*n* = 10) volunteers. Remarkably, following stimulation with Ty21a homogenate antigens, CD8+ T_RM_ produced significantly higher levels of net *S*. Typhi specific IFN-γ and IL-17A while CD8+CD69+CD103– T cells produced significantly higher levels of IFN-γ in Ty21a immunized volunteers compared to unvaccinated (Figures 8A,B).

**Figure 9:**
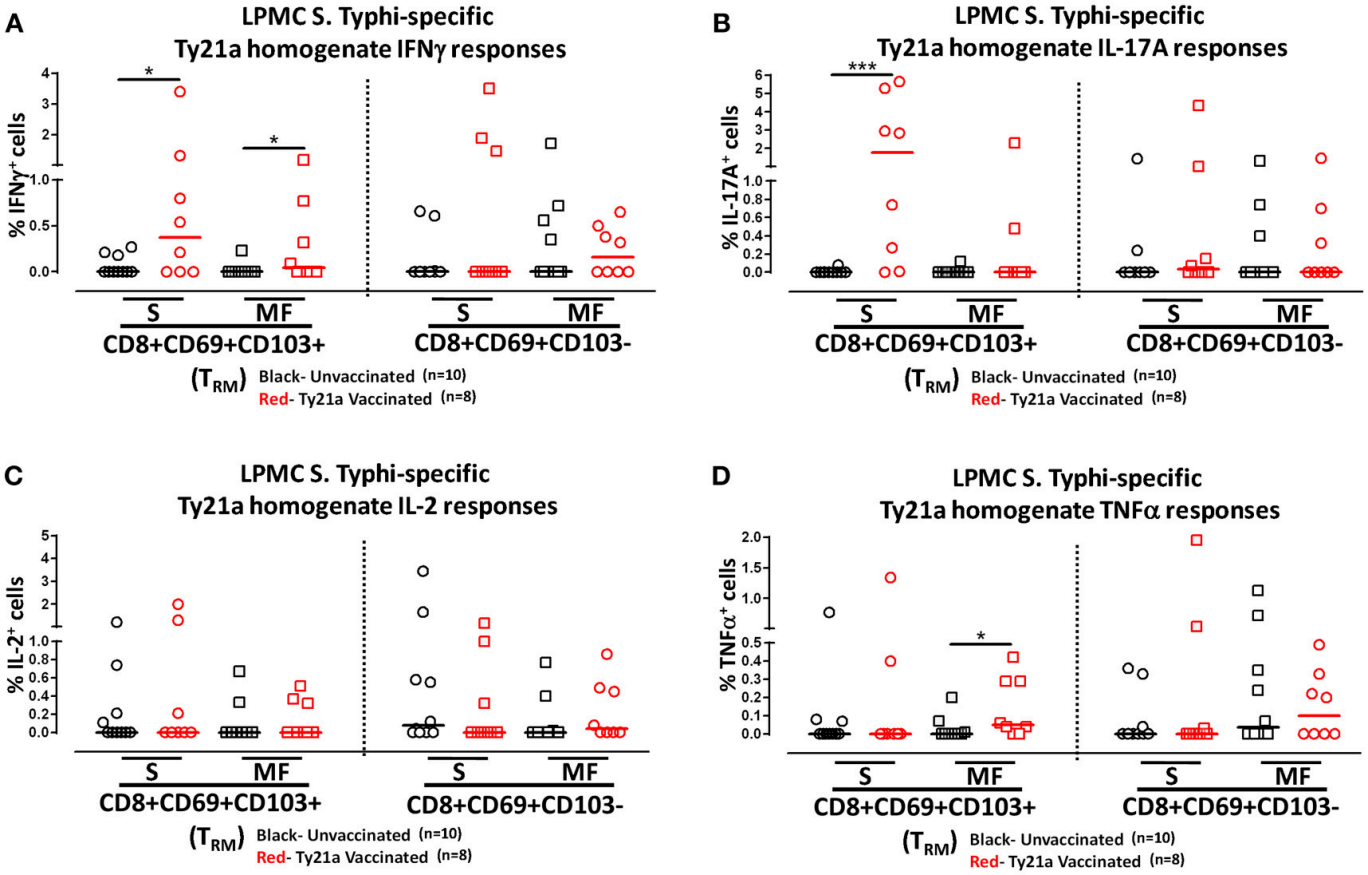
Characterization of multifunctional *S*. Typhi-specific responses to a Ty21a homogenate by terminal ileum LPMC CD8+ T_RM_ and CD8+CD69+CD103– T cell subsets in healthy adults following oral Ty21a-immunization. Terminal ileum LPMC CD8+ T_RM_ and CD8+CD69+CD103– T cells were stimulated with Ty21a homogenate (10 μg/mL) as described in section Materials and Methods. Net Ty21a homogenate (media subtracted) *S*. Typhi-specific CD8+ T_RM_ responses were calculated using the FCOM function of Winlist and stratified into multifunctional cells (MF) and single-positive cells (S). Comparison of TI LPMC CD8+ T_RM_ and CD8+CD69+CD103– Ty21a homogenate mediated *S*. Typhi-specific **(A)** INF-γ+; **(B)** IL-17A+; **(C)** IL-2+, and **(D)** TNF-α+ MF and S responses in Ty21a-vaccinated (*n* = 8; red symbols) and unvaccinated volunteers (*n* = 10; black symbols). Significant differences are shown (*P < 0.05; ***P < 0.0005). Horizontal bars (black and red) represent median values.

To further understand the differences in the elicited responses by the two subsets to soluble Ty21a homogenate antigens stimulation, we next investigated the multi-functionality of LPMC *S*. Typhi-specific CD8^+^ T_RM_ and CD8+CD69+CD103– T responses in Ty21a-vaccinated and unvaccinated volunteers following stimulation with Ty21a homogenate antigens as described above. First, we analyzed LPMC *S*. Typhi-specific CD8+ T_RM_ responses associated with IFN-γ production and observed that both CD8+ T_RM_ IFN-γ S or MF responsive cells were present in significantly (*P* < 0.05) higher percentages following Ty21a vaccination (Figure 9A). In contrast, no differences were observed in the frequencies of CD8+CD69+CD103– IFN-γ S or MF between Ty21a vaccinated volunteers and their unvaccinated counterparts (Figure 9A). Remarkably, we observed significant (*P* < 0.05) increases in LPMC *S*. Typhi-specific CD8+ T_RM_ IL-17A+ S but not MF following Ty21a vaccination (Figure 9B). No significant changes were observed in the levels of *S*. Typhi-specific CD8+CD69+CD103– IL-17A^+^ S and MF following Ty21a vaccination (Figure 9B). No differences were noted in *S*. Typhi-specific responses in IL-2+ S and MF (Figure 9C), and TNF-α+ S and MF (Figure 9D) except for a significant increase in CD8+ T_RM_ TNF-α MF response following Ty21a immunization (Figure 9D). In sum, LPMC CD8+ T_RM_ obtained from Ty21a-vaccinated volunteers respond primarily as IL-17A S effectors following stimulation with either soluble Ty21a homogenate antigens or *S*. Typhi-infected targets.

### Oral Ty21a Immunization Modulates Terminal Ileum IEL CD8+ T_RM_ *ex vivo*

We have observed that oral Ty21a immunization influences spontaneous production of cytokines by CD8+ T_RM_ in the lamina propria of the terminal ileum. We hypothesized that oral Ty21a immunization might similarly influence *ex vivo* responses without *in vitro* stimulation in the epithelial compartment of the human terminal ileum. To this end, we investigated the effect of oral Ty21a immunization on spontaneous production of cytokines by IEL CD8+ T_RM_. We first isolated TI IEL from biopsies obtained from volunteers who either received four doses of Ty21a or were unvaccinated and characterized CD8+ T_RM_ using CD69 and CD103 markers as shown in the gating strategy depicted in Figure S3. We observed that TI IEL CD8+ T_RM_ constitute the predominant population expressing high levels of CD69 and CD103. However, we observed differences in the frequencies of TI IEL CD8+ T_RM_ following Ty21a vaccination (Figures S3A,B). We also noted that following Ty21a vaccination, there were higher frequencies of TI IEL CD4+ T cells (Figure S3A). To determine whether there were differences in cell yields obtained from biopsies of Ty21a-vaccinated and unvaccinated volunteers, we compared the number of viable cells per mg of terminal ileum tissue and observed almost identical cell yields between the two groups (Figure S4A).

**Figure 10:**
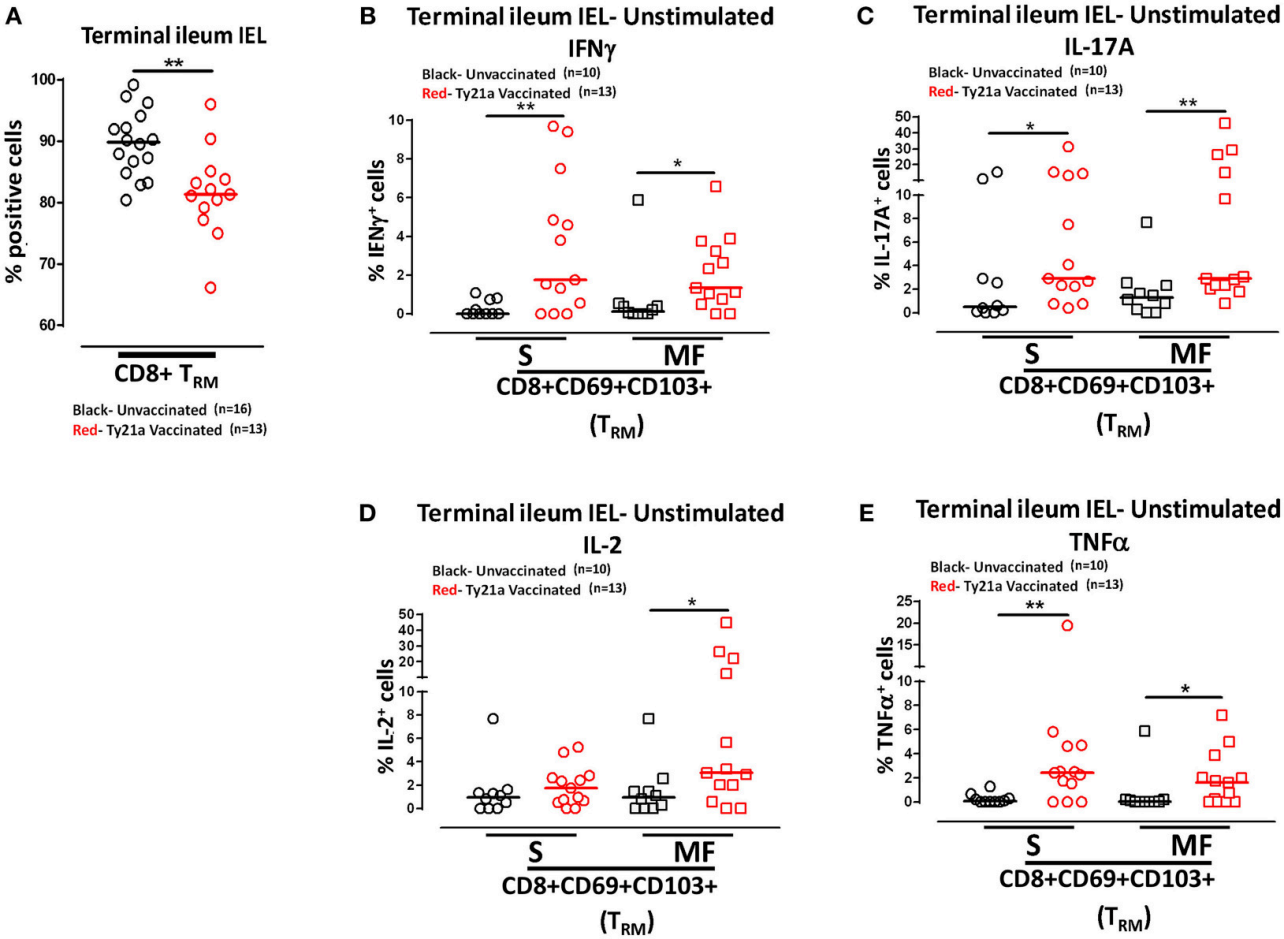
Effect of oral Ty21a-immunization on terminal ileum intraepithelial T lymphocytes (IEL) CD8+ T_RM_ frequencies and spontaneous cytokine production. **(A)** The frequencies of terminal ileum IEL CD8+ T_RM_ were determined and compared between TI IEL obtained from Ty21a-vaccinated (*n* = 13; red symbols) and unvaccinated volunteers (*n* = 16; black symbols). *Ex-vivo* unstimulated IEL CD8+ T_RM_ were cultured overnight and their cytokine (IFN-γ, IL-17A, IL-2, and TNF-α) production were determined by flow cytometry. Using the FCOM function of Winlist, IEL CD8+ T_RM_ responses were stratified into multifunctional cells (MF) and single-positive cells (S). Comparison of TI IEL CD8+ T_RM_ responses in **(B)** INF-γ+; **(C)** IL-17A+; **(D)** IL-2+, and **(E)** TNF-α+ MF and S in Ty21a-vaccinated (*n* = 13; red symbols) and unvaccinated volunteers (*n* = 10; black symbols). Significant differences are shown (*P < 0.05; **P < 0.005). Horizontal bars (black and red) represent median values.

Remarkably, the cumulative data indicated that the frequency of IEL CD8+ T_RM_ obtained from Ty21a vaccinated individuals were significantly lower than those from unvaccinated volunteers (Figure 10A). We next measured and compared the frequency of single (S) and multifunctional (MF) producing cytokines (IFN-γ, IL-17A, IL-2, and TNF-α) in IEL CD8+ T_RM_ obtained from Ty21a vaccinated (*n* = 13) and unvaccinated (*n* = 10) volunteers following unstimulated overnight cultures. We found that *ex-vivo* unstimulated IEL CD8+ T_RM_ obtained from Ty21a immunized volunteers have significantly higher levels of IFN-γ S and MF than unimmunized volunteers (Figure 10B). Similarly, we found that IEL CD8+ T_RM_ produced spontaneously significantly higher IL-17A+ S and MF (Figure 10C) and TNF-α+ S and MF (Figure 10E) following Ty21a immunization. We also observed that IEL exhibit significant differences in the frequencies of IL-2+ MF in IEL CD8+ T_RM_ following Ty21a vaccination (Figure 10D).

### Oral Ty21a-Immunization Elicits Terminal Ileum IEL *S*. Typhi Responsive CD8^+^ T_RM_

**Figure 11:**
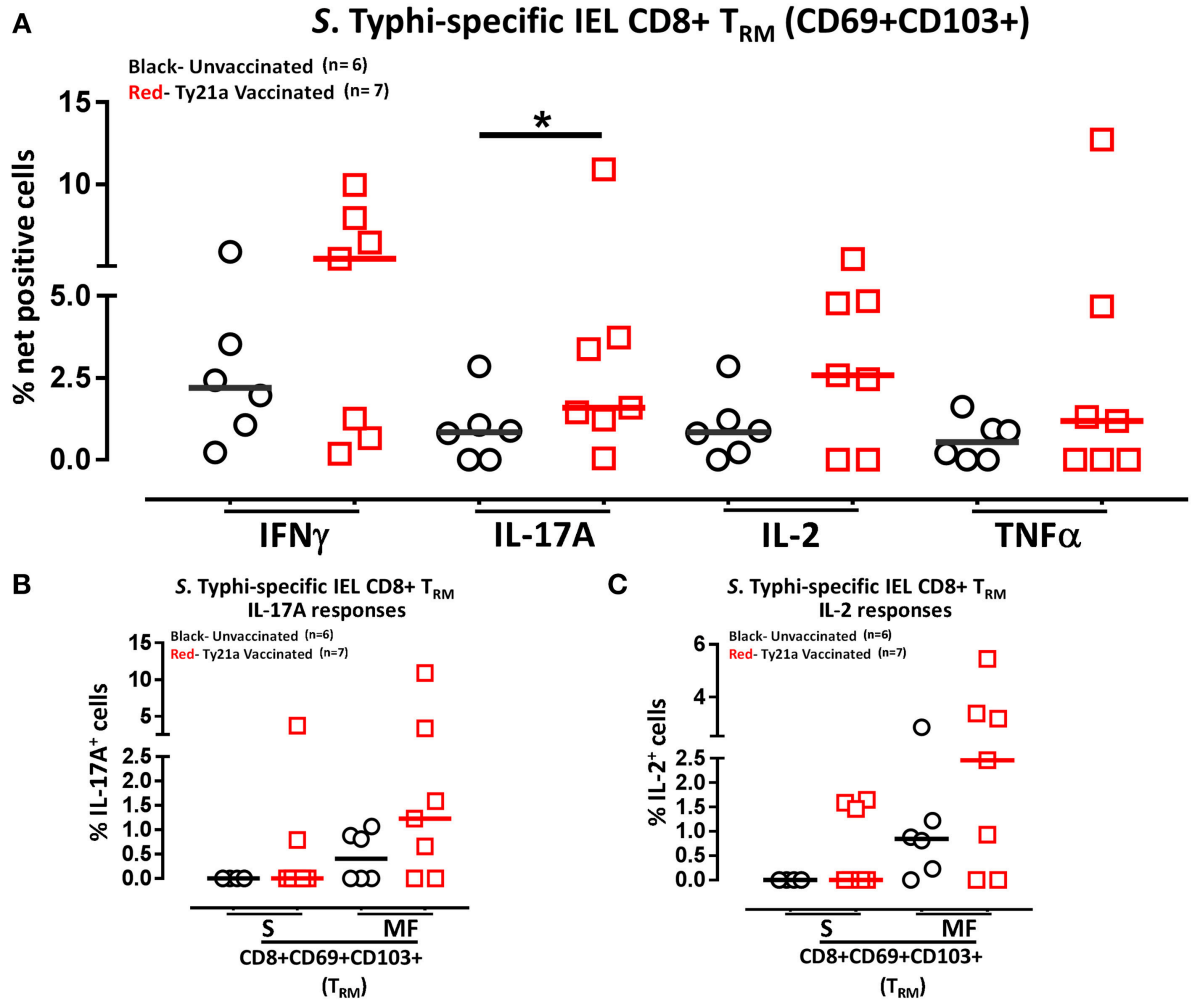
*S*. Typhi-specific responses of terminal ileum IEL CD8+ T_RM_ in healthy adults following oral Ty21a-immunization. **(A)** The net percentages of *S*. Typhi-specific responses (IFN-γ, IL-17A, IL-2, and TNF-α) of terminal ileum IEL CD8+ T_RM_ were determined and compared between TI IEL obtained from Ty21a-vaccinated (*n* = 7; red symbols) and unvaccinated volunteers (*n* = 6; black symbols). Using the FCOM function of Winlist, IEL CD8+ T_RM_ responses were stratified into multifunctional cells (MF) and single-positive cells (S). Comparison of *S*. Typhi-specific TI IEL CD8+ T_RM_ responses in **(B)** IL-17A+; **(C)** IL-2+, MF and S in Ty21a-vaccinated (*n* = 7; red symbols) and unvaccinated volunteers (*n* = 6; black symbols). Significant differences are shown (*P < 0.05). Horizontal bars (black and red) represent median values.

Above we described our observations on the presence of *S*. Typhi responsive CD8+ T_RM_ in the terminal ileum lamina propria following Ty21a immunization and that CD8+ T_RM_ express high levels of CD103, integrin αE, which is a ligand to E-cadherin found on epithelial cells. Thus, it was reasonable to hypothesize that these CD8+ T_RM_ are poised to migrate to the epithelium and contribute to *S*. Typhi immunity as IEL. To test this hypothesis, we evaluated the ability of IEL CD8+ T_RM_ obtained from TI biopsies of Ty21-vaccinated (*n* = 7) and unvaccinated (*n* = 6) volunteers to elicit *S*. Typhi-specific responses following stimulation with autologous *S*. Typhi-infected and uninfected targets cells. Net percentages of positive cells were calculated as % of *S*. Typhi-infected EBV-B responses—% of uninfected EBV-B responses as previously described. Cumulative data of IEL *S*. Typhi-specific CD8+ T_RM_ responses expressed as net percentage of positive cells are shown in Figure 11A. Remarkably, IEL CD8+ T_RM_ exhibited significantly (*p* < 0.05) higher *S*. Typhi-specific IL-17A responses in Ty21a vaccinated volunteers than their unvaccinated counterparts (Figure 11A). We also noted that IEL CD8+ T_RM_ appear to show some increases in the production of IFN-γ and IL-2 cytokine following Ty21a-vaccination in some volunteers, which, as a group, did not reach statistical significance (Figure 11A). This is, to our knowledge, the first demonstration of local *S*. Typhi-specific CD8+ T_RM_ responses in human epithelium (as IEL) and further suggests that CD8+ T_RM_ are acting primarily as T_C_17 at the local mucosa following oral Ty21a-immunization.

We have showed above that terminal ileum LPMC CD8+ T_RM_ respond to *S*. Typhi-infected targets by secreting either single or multiple cytokines simultaneously. However, it is unknown whether IEL *S*. Typhi-specific CD8+ T_RM_ cells responses exhibit similar multi-functionality. This approach could help in better understanding the local immunity to *S*. Typhi responses. Thus, we next investigated the multi-functionality of IEL *S*. Typhi-specific CD8+ T_RM_ responses in Ty21a-vaccinated and unvaccinated volunteers using the Winlist FCOM function. We analyzed IEL *S*. Typhi-specific CD8+ T_RM_ responses for IL-17A (Figure 11B), IL-2 (Figure 11C), IFNγ (Figure S4B), and TNF-α (Figure S4C) production. We observed that the responses were mostly MF, with some individuals showing higher IL-17A, IL-2, IFN-γ, and TNF-α production in S and/or MF cells following Ty21a vaccination, but which, as a group, did not reach statistical significance, likely due to the relatively limited number of participants evaluated.

### Relationship of Multifunctional *S*. Typhi-Specific CD8+ T_RM_ Responses Between LPMC and IEL

Because CD8+ T_RM_ are elicited specifically following oral Ty21a immunization in both the lamina propria and epithelium compartments, albeit differently, we explored the relationship between the generation of *S*. Typhi specific immune responses between LPMC and IEL in an individual by individual basis. To this end we performed Spearman correlation tests between LPMC and IEL CD8+ T_RM_ S and MF responses in both Ty21a vaccinated and unvaccinated volunteers. We observed that in unvaccinated volunteers, the frequencies of LPMC CD8+ T_RM_ S and MF (IFN-γ, IL-17A IL-2, and TNF-α) responses were not correlated to their IEL counterparts (Table S2). However, following Ty21a vaccination, the frequencies of LPMC CD8+ T_RM_ MF (IL-17A and IL-2) responses, but not S, were significantly correlated to their IEL counterparts (Table S2). In addition, we analyzed the multifunctional CD8+ T_RM_ *S*. Typhi-specific subtypes elicited following Ty21a immunization in terminal ileum LPMC and IEL (Figure S5). We observed that the two major *S*. Typhi MF effector subtypes in LPMC CD8+ T_RM_ significantly elicited were IL-17A+ IL-2+ and IFN-γ+ TNF-α+ double producers following Ty21a immunization (Figure S5). Similar comparisons in IEL CD8+ T_RM_ revealed analogous trends, but they did not reach statistical significance following Ty21a immunization (Figure S5).

**Figure 12:**
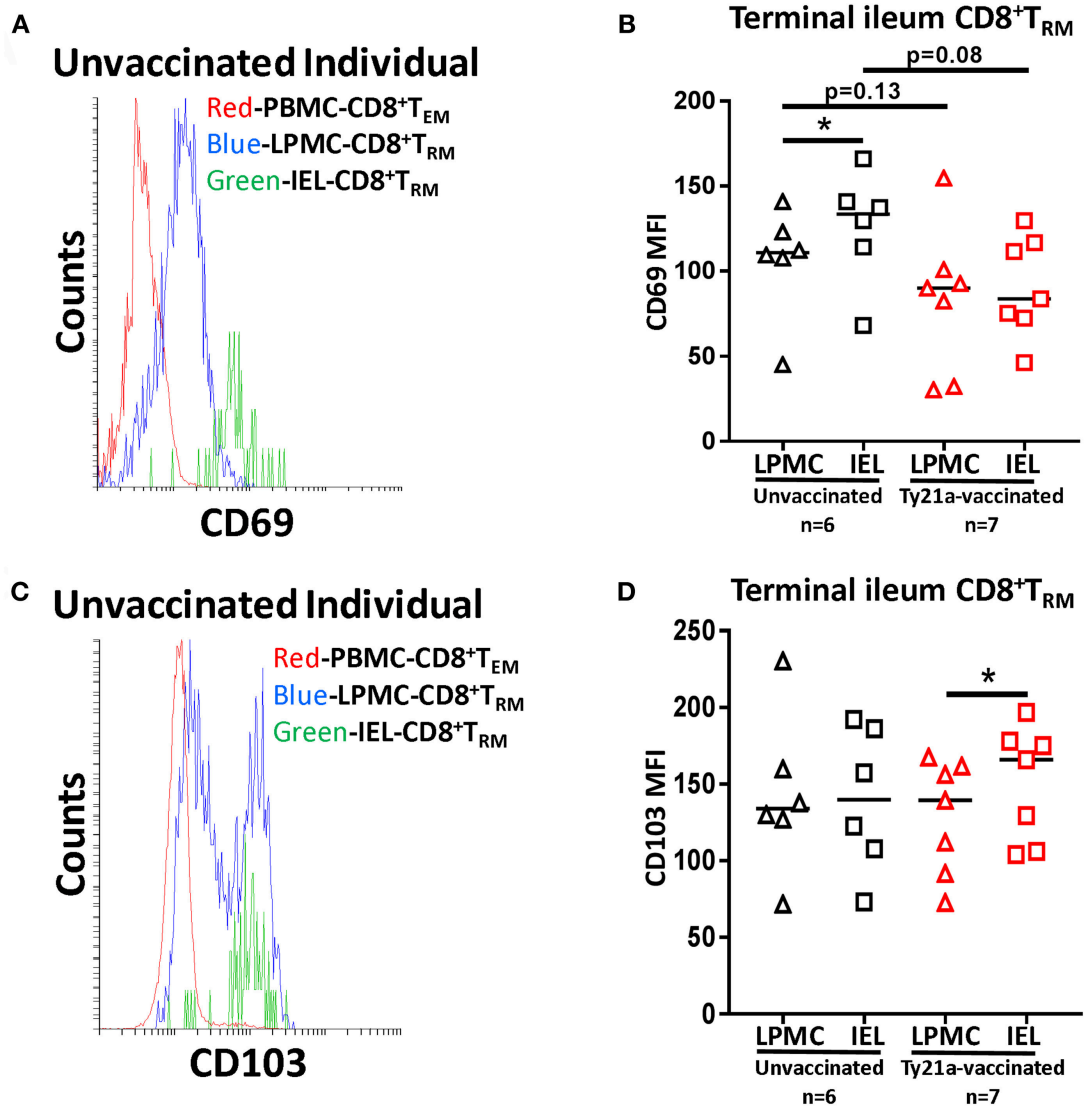
Terminal ileum IEL CD8+ T_RM_ expressed differential levels of CD69 and CD103 expression than their LPMC counterparts following Ty21a-immunization. The mean fluorescence intensity (MFI) of **(A)** CD69 and **(C)** CD103 were determined in CD8+ T_RM_ obtained from PBMC, LPMC and IEL of a representative unvaccinated volunteer. Comparison of **(B)** CD69 and **(D)** CD103 MFI expression on IEL and LPMC CD8+ T_RM_ obtained from Ty21a-vaccinated (red; *n* = 7) and unvaccinated (black; *n* = 6) volunteers were assessed with significant differences shown (*P < 0.05). Horizontal black bars represent median values.

To further understand the relationship between LPMC and IEL, we measured the mean fluorescence intensity (MFI) of CD69 and CD103 expressed on CD8+ T_RM_ from each individual volunteer following Ty21a immunization (Figure 12). As shown in the cytogram of the representative unvaccinated volunteer, CD69 is expressed at a higher level on CD8+ T_RM_ obtained from IEL (green line) than in LPMC (blue line) (Figure 12A). As expected, CD8+ T_EM_ obtained from PBMC (red line) expressed much lower levels of CD69 as compared to LPMC (Figure 12A). We next compared the level of CD69 between IEL and LPMC CD8+ T_RM_ obtained from either Ty21a vaccinated (*n* = 7) or unvaccinated (*n* = 6) volunteers. Cumulative data shows that in unvaccinated volunteers, the levels of CD69 expression on CD8+ T_RM_ were significantly (*P* < 0.05) higher in IEL than in LPMC (Figure 12B). In contrast, following Ty21a vaccination, no trends were observed in CD69 MFI levels between these cell subsets (Figure 12B). Of note, we observed a trend to lower levels of CD69 expression in both IEL and LPMC CD8+ T_RM_ in vaccinated as compared to unvaccinated participants (Figure 12B). We also determined the levels of CD103 expression as shown by an unvaccinated representative volunteer (Figure 12C) where the level of expression of CD103 on CD8+ T_RM_ was shown to be similar between IEL (green line) and LPMC (blue line) but, as expected, absent in PBMC CD8+ T_EM_ (red line) (Figure 12C). We next compared between the two groups of individuals. Cumulative data indicated that in unvaccinated volunteers, the level of CD103 expression on CD8+ T_RM_ was similar between IEL and LPMC (Figure 12D). In contrast, following Ty21a vaccination, we observed significantly higher expression of CD103 in IEL than in LPMC CD8+ T_RM_ (Figure 12D).

### Relationship of Mucosal (LPMC and IEL) CD8+ T_RM_ Responses to Systemic (PBMC) *S*. Typhi-Specific CD8+T_EM_

It was previously reported that oral Ty21a immunization elicits both LPMC and PBMC CD8+ T_EM_ (32), and in this study we demonstrated that CD8+ T_RM_ are elicited in both LPMC and IEL following Ty21a immunization. To enable the pairwise comparison of *S*. Typhi-specific responses in the various compartments, we sampled simultaneously PBMC and terminal ileum biopsies in each volunteer. We hypothesized that the induction of systemic *S*. Typhi responsive CD8+T_EM_ would differ in quantity and quality to those elicited in CD8+ T_RM_ in mucosal tissues (LPMC and IEL) following Ty21a immunization. To explore this hypothesis, we investigated the relationship between the generations of *S*. Typhi-specific immune responses between PBMC CD8+ T_EM_ and LPMC CD8+ T_RM_ on an individual by individual basis. To this end we performed Spearman correlation tests between PBMC CD8+ T_EM_ and LPMC CD8+ T_RM_ S and MF responses in Ty21a vaccinated (*n* = 9) volunteers. We observed that following Ty21a vaccination, the frequencies of PBMC CD8+ T_EM_ S (TNF-α) responses, but not MF, were significantly negatively correlated to their LPMC counterparts (Table S3), the level of PBMC CD8+ T_EM_ S (TNF-α) responses being higher that their LPMC counterparts. Trends toward positive correlations were observed in S and MF IFN-γ and MF IL-17A production (Table S3). We also explored, using a similar approach, the relationship between the generation of *S*. Typhi-specific immune responses between PBMC CD8+ T_EM_ and IEL CD8+ T_RM_ S and MF responses on an individual by individual basis in Ty21a vaccinated volunteers. We observed that following Ty21a vaccination, the frequencies of PBMC CD8+ T_EM_ MF (IL-2) responses, but not S, were significantly negatively correlated to their IEL counterparts (Table S3), the frequency of IEL CD8+ T_RM_ IL-2 MF being higher than their PBMC CD8+ T_EM_ counterparts (Table S3).

## Discussion

The ability of tissue resident memory T cells (T_RM_) to rapidly mount strong protective immunity to site-specific infection has increasingly become an important focus to evaluate T cell–mediated responses as part of vaccine development efforts. Here, we determined the effect of oral immunization with the attenuated oral typhoid vaccine Ty21a on human terminal ileum LPMC and IEL CD8+ T_RM_. We established that immunization results in increased spontaneous cytokine production. Importantly, we also observed cytokine production in response to *S*. Typhi-responsive CD8+ T_RM_, which were further increased following Ty21a immunization. These increases following oral Ty21a immunization were primarily in Tc17 in both terminal ileum mucosal compartments, i.e., lamina propria (LPMC) and epithelium (IEL). Moreover, we uncovered that LPMC CD8+CD69+CD103– T cells subsets contributed significantly to *S*. Typhi-specific IFN-γ, IL-17A, and IL-2 responses following Ty21a immunization. Our results also showed that oral Ty21a immunization influence the ability of LPMC to respond to stimulation through the T cell receptor and the CD28 co-stimulatory molecule using α-CD3/CD28 beads. Taken together, these results contribute novel information of the effects of oral vaccination on terminal ileum mucosal responses in humans which could have significant implications in vaccine design and development.

Enteric pathogens (e.g., *S*. Typhi) or oral Ty21a vaccine strain can actively enter intestinal epithelium at the site of infection (e.g., TI) through a variety of mechanisms including epithelial and M cells invasion (40, 41). The assumption is that following invasion, bacterial antigens activate the innate immune system and are presented to T_M_ cells including T_RM_ by antigen presenting cells (APC) in the lamina propria and the epithelium eliciting protective adaptive effector immune responses. Given the great difficulties associated with obtaining cells from terminal ileum, the preferred site of infection of the human-restricted *S*. Typhi bacteria in humans, very limited information is available on the responses in the human gut microenvironment. Using this unique model involving oral immunization with an attenuated typhoid vaccine in humans, we observed that vaccination results in increases in the spontaneous production of significantly higher levels of cytokines (IFN-γ and TNF-α) as single producing effectors in both LPMC CD8+ T_RM_ and CD8+CD69+CD103– T cells. However, increases in the spontaneous production of IL-17A following Ty21a vaccination were only observed in LPMC CD8+ T_RM_ S but not LPMC CD8+CD69+CD103– T cells, which had significant decreased in spontaneous IL-17A production. Overall, these results indicate that oral Ty21a immunization influence the ability of LPMC CD8+ T_RM_ S to become Tc1 and Tc17 effectors, while LPMC CD8+CD69+CD103– T cells exhibit Tc1 effector characteristics. Some of these effects were also observed following stimulation with α-CD3/CD28 stimulation. This may be explained by the fact that although LPMC is a relatively homogenous population expressing CD8, CD103, and CD69, they can be composed of multiple cell subsets which differ in their requirements for activation signals and/or on the specific cognate antigens they recognize (42–44). Thus, the spontaneous production of cytokines by LPMC CD8+ T_RM_ could be derived from LPMC CD8+ T_RM_ and a variety of other resident LPMC T cells, such as mucosal associated invariant T cells (MAIT) and natural killer T cells (NKT) following oral Ty21a immunization (45). Further studies will be required to fully understand the contribution of various cell subsets to the observed increase in spontaneous cytokine production following immunization. Importantly, these novel results show that immunization with bacterial oral vaccines may have immunomodulatory effects beyond those that are specific for the vaccine being administered.

We next provided evidence that oral Ty21a-immunization elicits significant LPMC CD8+ T_RM_ *S*. Typhi-specific responses producing primarily IL-17A. These antigen-specific responses were mostly observed as single-producing effectors suggesting that *S*. Typhi-responsive CD8+ T_RM_ S are primarily Tc17. Remarkably LPMC CD8+CD69+CD103– T cells were elicited to produce significantly higher *S*. Typhi-specific IFN-γ, IL-17A, and IL-2 levels following Ty21a vaccination. These results suggest a dichotomy in *S*. Typhi-specific responses in TI following Ty21a immunization whereby LPMC CD8+ T_RM_ are mostly Tc17 (IL-17A), whilst CD8+CD69+CD103– T cells produced multiple cytokines associated with both Tc1 (IFN-γ and IL-2) and Tc17 (IL-17), a set of effector immune responses well-suited for protection against intracellular pathogens.

We also analyzed whether CD8+ T_RM_ and CD8+CD69+CD103– T LPMC responses following Ty21a immunization depend on the antigen used for *in vitro* stimulation. To this end we used *S*. Typhi-infected targets (an efficient CD8+ T stimulation) and Ty21a homogenate antigens (a less efficient CD8+ T stimulation) (46) to stimulate CD8+ T_RM_ and CD8+CD69+CD103– T cells. Interestingly, our results indicate that following stimulation with Ty21a homogenate antigens, LPMC CD8+ T_RM_ primarily produced not only significant *S*. Typhi-specific IL-17A **S** responses, but also significant IFN-γ MF and TNF-α MF T_RM_ in the vaccinated group compared to the unvaccinated group. The latter results were not observed with *S*. Typhi-infected targets stimulation, which largely display *S*. Typhi antigens in the context of MHC-I molecules. In contrast, following stimulation with the Ty21a homogenate, LPMC CD8+CD69+CD103– T cells showed no increase in cytokines production by MF or S in the Ty21a vaccinated group. These results suggest that LPMC CD8+ T_RM_ are more versatile in recognizing and responding to *S*. Typhi antigens primarily as Tc17 effectors than CD8+CD69+CD103– T cells. Of note, we recently reported that Ty21a immunization also elicits the induction of *S*. Typhi-specific CD4+ T_M_ to secrete cytokines following *in vitro* stimulation, indicating that multiple effector T cell responses are concomitantly induced (47). Future studies looking at the activation requirements of CD4 and CD8 populations in human tissues would be crucial to better understand these phenomena to advance mucosal vaccines targeting the induction of T cell mediated immunity.

CD8+ T_RM_ express abundant levels of integrin αE (CD103) which forms a heterodimer with integrin β7 and recognizes E-cadherin expressed on the intestinal epithelial cells (48). Intraepithelial lymphocytes (IEL) have been proposed as being an important cell subset involved in immune responses at mucosal surfaces (49, 50). Thus, we deemed important to also focus on this important cell population following oral immunization in the present human oral immunization model. We describe, for the first time, that oral Ty21a immunization elicits activated IEL CD8+ T_RM_ responses with significantly increased spontaneous production of cytokines (IFN-γ, IL-17A, IL-2, and TNF-α), both as single cytokine producing and multifunctional effectors. These results are similar to the spontaneous cytokine production described for LPMC populations above and can also represent the combined response of multiple cell subsets (e.g., classical CD8+ T_RM_, MAIT, NKT) which are activated to spontaneously produce cytokines following oral immunization.

Regarding *S*. Typhi-specific cytokine production, we found that oral Ty21a-immunization elicits significant IEL CD8+ T_RM_ producing primarily IL-17A, although a few volunteers also exhibited strong IL-2, IFN-γ, and TNF-α production. These antigen-specific responses were observed both as single-producing and multifunctional effectors and may represent IEL derived from conventional CD8αβ T cells recognizing *S*. Typhi antigens presented in the context of MHC class I molecules which have homed to the intestinal epithelium following recognition of their cognate antigen in the intestine (43, 51). Taken together, our findings suggest that adaptive IEL CD8+ T_RM_ elicited by oral Ty21a immunization as both, Tc1 and Tc17 subsets. In future studies it will be essential to understand the activation properties these IEL T_RM_ cells to gain further insights into the mechanism regulating local gut immunity in humans.

Interestingly, comparisons of CD8+ T_RM_ located in different compartments (e.g., lamina propria vs. epithelium) indicate a positive relationship in *S*. Typhi-specific CD8+ T_RM_ MF responses between LPMC and IEL following Ty21a immunization. In addition, we also observed that there are dominant MF subtypes (IL-17A+ IL-2+ and IFN-γ+ TNF-α+ double producers) elicited following Ty21a immunization and that these subtypes were present in LPMC, although they were also observed in IEL from a few volunteers. Taken together, these results suggest that CD8+ T_RM_ MF may be better poised to shuttle between LPMC and IEL, and, therefore, they are likely to play a major effector function in the epithelium. This observation further substantiate our recent report that *S*. Typhi-specific CD8^+^ MF responses correlate with protection against typhoid disease onset in a *S*. Typhi human challenged model (37). Thus, MF *S*. Typhi-specific responses elicited int the terminal ileum could be a key factor in protection against typhoid disease. These results are important because of mounting evidence that the MF characteristics of immune responses are significant determinants of effective immunity against pathogens. In fact, the induction of MF responses at higher magnitudes than single cytokine-secreting cells have been shown to be important, including in some cases being associated with protection, in various disease and vaccine models, including HIV, Cytomegalovirus, vaccinia, EBV, *Leishmania, M. tuberculosis*, and Ebola (52–59). Thus, it is likely that the development of future generations of improved oral *Salmonella* vaccines need to prioritize the induction of MF CD8+ T_EM_ cells in the mucosal microenvironment.

Finally, we also compared the systemic (PBMC CD8+ T_EM_) and mucosal (LPMC and IEL) CD8+ T_RM_ compartments. Interestingly, these comparisons indicated that PBMC CD8+ T_EM_ MF and LPMC CD8+ T_RM_ MF have a positive relationship (positive Spearman Rho values), although this correlation did not reach statistical significance likely due to the relatively small number of individuals evaluated. These observations confirm and extend our previous findings of significant positive correlations between LPMC and PBMC CD8+ T_EM_ MF cells (32).

In closing, we provide the first evidence of the induction of increased spontaneous and *S*. Typhi-specific cytokine production by CD8+ T_RM_ in the lamina propria and intestinal epithelium of the human terminal ileum mucosa following oral immunization with Ty21a. These results contribute novel insights into our understanding of the generation of gut local immunity in humans following immunization with oral attenuated bacteria and suggest that CD8+ T_RM_ play a key role in protection following immunization and/or infection with *S*. Typhi.

## Ethics Statement

Volunteers with no history of typhoid fever were enrolled from the Baltimore-Washington metropolitan area and University of Maryland, Baltimore campus. All terminal ileum biopsies and blood specimens were obtained from healthy volunteers undergoing routine colonoscopy who participated in the clinical protocol (# HP 00056321) approved by the University of Maryland Institutional Review Board (IRB). This study was carried out following the ethical standards in accordance with the Declaration of Helsinki. After the purpose and risk of the study was explained to the volunteers, written informed consent was obtained from the volunteers.

## Author Contributions

JB performed the experiments, contributed to study design, acquisition of data, analysis, and drafting of the manuscript. SP and EG performed endoscopies, obtained terminal ileum biopsies and reviewed the manuscript. RB contributed to patient recruitment, collection of peripheral blood mononuclear cells, and terminal ileum biopsies, and reviewed the manuscript. BG performed endoscopies, obtained terminal ileum biopsies, and reviewed the manuscript. MS designed the study, supervised the work and drafted the manuscript.

### Conflict of Interest Statement

The authors declare that the research was conducted in the absence of any commercial or financial relationships that could be construed as a potential conflict of interest.

## Supplementary Material

Figure S1 Spontaneous and *S*. Typhi-specific responses in LPMC CD8+ T cell subsets isolated from terminal ileum from an unvaccinated representative volunteer. **(A)** CD8+ T_RM_ and **(B)** CD8+CD69+CD103– T cells were stimulated with non-infected or *S*. Typhi-infected autologous EBV-B cells and the produced cytokines (IFNγ, IL-17A, IL-2, and TNFα) determined. Anti (α)-CD3/CD28 stimulation was used as a positive control in both subsets while unstimulated LPMC CD8+ T_RM_ or CD8+CD69+CD103– T cells alone was used as a negative control. In addition, unstained cell controls were used to place the delimiters for LPMC auto-fluorescence. The percentage of positive cells in the gated regions is shown above the corresponding black boxes.

Figure S2 Oral Ty21a-immunization induces differential activation on terminal ileum LPMC CD8+ T_RM_ subsets single and multifunctional cytokine responses following anti-CD3/CD28 stimulation. Following anti-CD3/CD28 stimulation, CD8+ T_RM_ and CD8+CD69+CD103– T cytokine responses were stratified into multifunctional cells (MF) and single-positive cells (S). Comparison of TI LPMC CD8+ T_RM_ and CD8+CD69+CD103– T cells responses in **(A)** INF-γ+; **(B)** IL-17A+; **(C)** IL-2+, and **(D)** TNF-α+ MF and S in Ty21a-vaccinated (*n* = 14; red symbols) and unvaccinated volunteers (*n* = 20; black symbols) were determined with significant differences shown (^*^*P* < 0.05; ^**^*P* < 0.005; ^***^*P* < 0.0005). Black lines: significant differences between Ty21a vaccinated and unvaccinated volunteers. Blue lines: significant differences between S and MF. Green lines: significant differences between CD8+ T_RM_ and CD8+CD69+CD103– T cell responses. Horizontal bars (black and red) represent median values.

Figure S3 Gating Strategy for identifying terminal ileum IEL CD8^+^ tissue-resident T memory cell subsets (T_RM_). Terminal ileum intraepithelial T lymphocytes were isolated and IEL CD8^+^ tissue resident T memory cell (T_RM_) identified using CD69 and CD103 markers. Shown are representative volunteers. **(A)** Ty21a vaccinated. **(B)** unvaccinated.

Figure S4 Absolute number of viable terminal ileum cells and *S*. Typhi-specific responses of terminal ileum IEL CD8+ T_RM_ in healthy adults following oral Ty21a-immunization. **(A)** The number of freshly isolated terminal ileum IEL obtained from biopsies of Ty21a-vaccinated (*n* = 13; red symbols) and unvaccinated (*n* = 16; black symbols) volunteers were compared. The net percentages of *S*. Typhi-specific responses (IFN-γ, IL-17A, IL-2, and TNF-α) of terminal ileum IEL CD8+ T_RM_ were determined and compared between TI IEL obtained from Ty21a-vaccinated (*n* = 7; red symbols) and unvaccinated volunteers (*n* = 6; black symbols). Using the FCOM function of Winlist, IEL CD8+ T_RM_ responses were stratified into multifunctional cells (MF) and single-positive cells (S). Comparison of *S*. Typhi-specific TI IEL CD8+ T_RM_ responses in **(B)** IFN-γ+; **(C)** TNF-α+ Horizontal bars (black and red) represent median values.

Figure S5 Multifunctional *S*. Typhi responsive CD8+ T_RM_ responses in terminal ileum LPMC and IEL in Ty21a-vaccinated and unvaccinated volunteers. Induction of multiple cytokines (IFN-γ, IL-17A, IL-2, and TNF-α) in terminal ileum LPMC and IEL following stimulation with *S*. Typhi-infected EBV-B cells. Data were analyzed using FCOM function of Winlist and six (out of 16) significant combinations are represented in the figure with double, triple and quadruple responses with significant differences (^*^*P* < 0.05; ^**^*P* < 0.005) between vaccinated and unvaccinated volunteers shown. Horizontal bars (black and red) represent median values.

Table S1 Demographics of the participants included in this study.

Table S2 Spearman correlation analysis between LPMC and IEL CD8+T_RM_ *S*. Typhi responses in unvaccinated and Ty21a-vaccinated volunteers.

Table S3 Spearman correlation analysis between mucosal (LPMC and IEL) CD8+ T_RM_ and PBMC T_EM_ *S*. Typhi responses in Ty21a-vaccinated volunteers.
