# Supplementary figures and images for: Attenuated Oral Typhoid Vaccine Ty21a Elicits Lamina Propria and Intra-Epithelial Lymphocyte Tissue-Resident Effector Memory CD8 T Responses in the Human Terminal Ileum
Source: Front Immunol. 2019 Mar 14;10:424. doi: 10.3389/fimmu.2019.00424 (PMC6426796; doi:10.3389/fimmu.2019.00424)

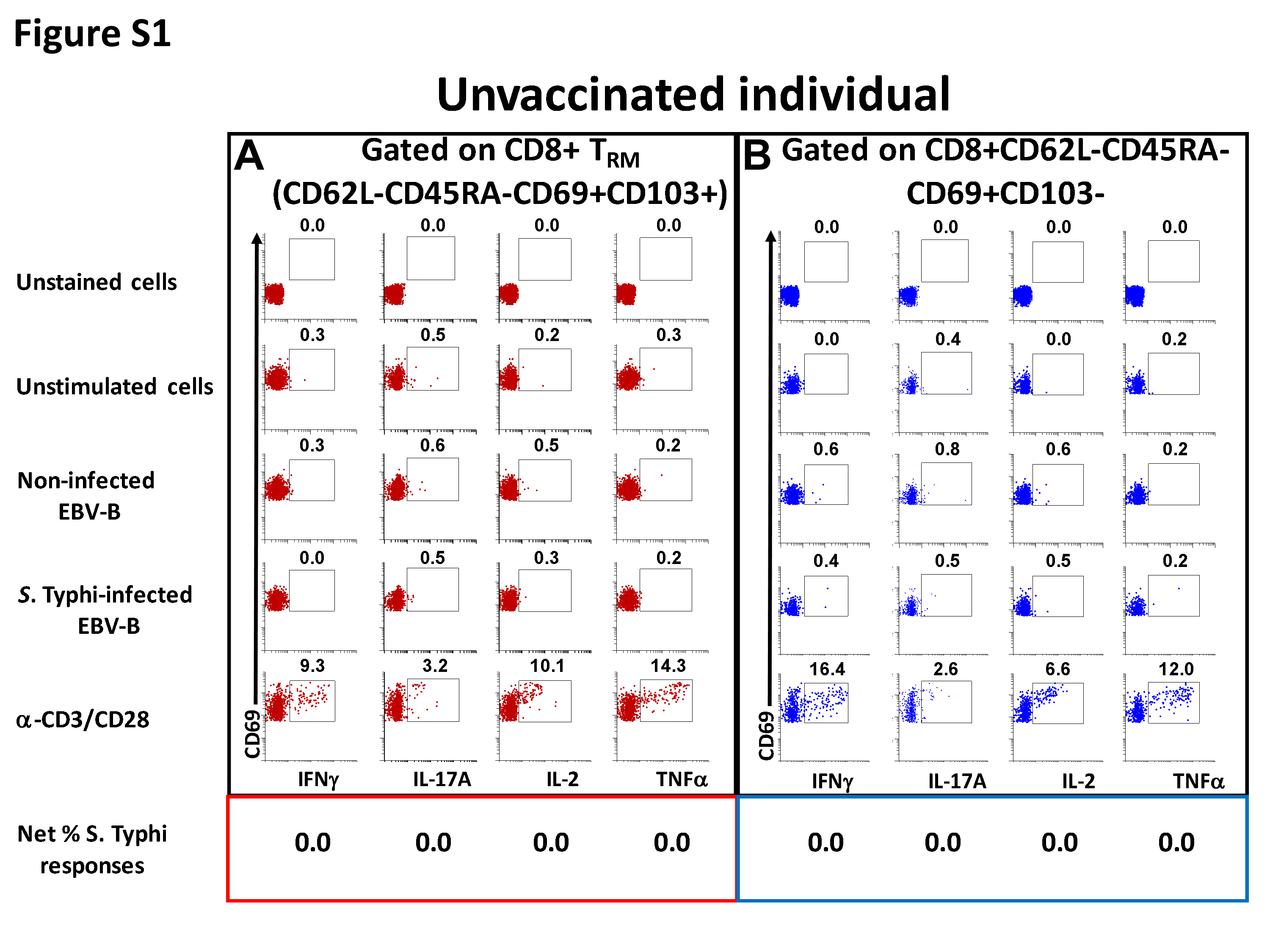

Supplement: Figure S1 — Spontaneous and S. Typhi-specific responses in LPMC CD8+ T cell subsets isolated from terminal ileum from an unvaccinated representative volunteer. (A) CD8+ TRM and (B) CD8+CD69+CD103– T cells were stimulated with non-infected or S. Typhi-infected autologous EBV-B cells and the produced cytokines (IFNγ, IL-17A, IL-2, and TNFα) determined. Anti (α)-CD3/CD28 stimulation was used as a positive control in both subsets while unstimulated LPMC CD8+ TRM or CD8+CD69+CD103– T cells alone was used as a negative control. In addition, unstained cell controls were used to place the delimiters for LPMC auto-fluorescence. The percentage of positive cells in the gated regions is shown above the corresponding black boxes. [file Image_1.tif]

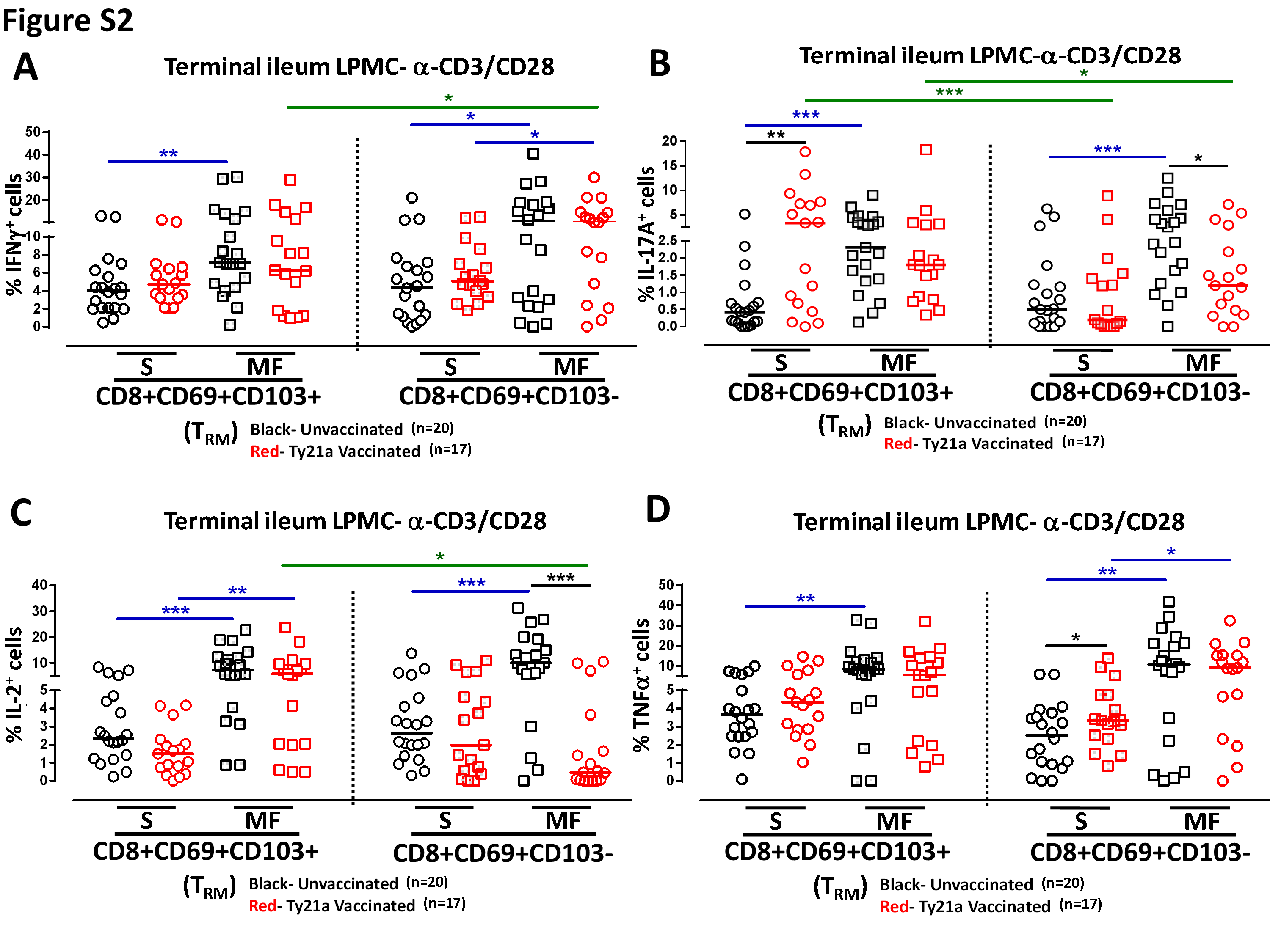

Supplement: Figure S2 — Oral Ty21a-immunization induces differential activation on terminal ileum LPMC CD8+ TRM subsets single and multifunctional cytokine responses following anti-CD3/CD28 stimulation. Following anti-CD3/CD28 stimulation, CD8+ TRM and CD8+CD69+CD103– T cytokine responses were stratified into multifunctional cells (MF) and single-positive cells (S). Comparison of TI LPMC CD8+ TRM and CD8+CD69+CD103– T cells responses in (A) INF-γ+; (B) IL-17A+; (C) IL-2+, and (D) TNF-α+ MF and S in Ty21a-vaccinated (n = 14; red symbols) and unvaccinated volunteers (n = 20; black symbols) were determined with significant differences shown (*P < 0.05; **P < 0.005; ***P < 0.0005). Black lines: significant differences between Ty21a vaccinated and unvaccinated volunteers. Blue lines: significant differences between S and MF. Green lines: significant differences between CD8+ TRM and CD8+CD69+CD103– T cell responses. Horizontal bars (black and red) represent median values. [file Image_2.TIF]

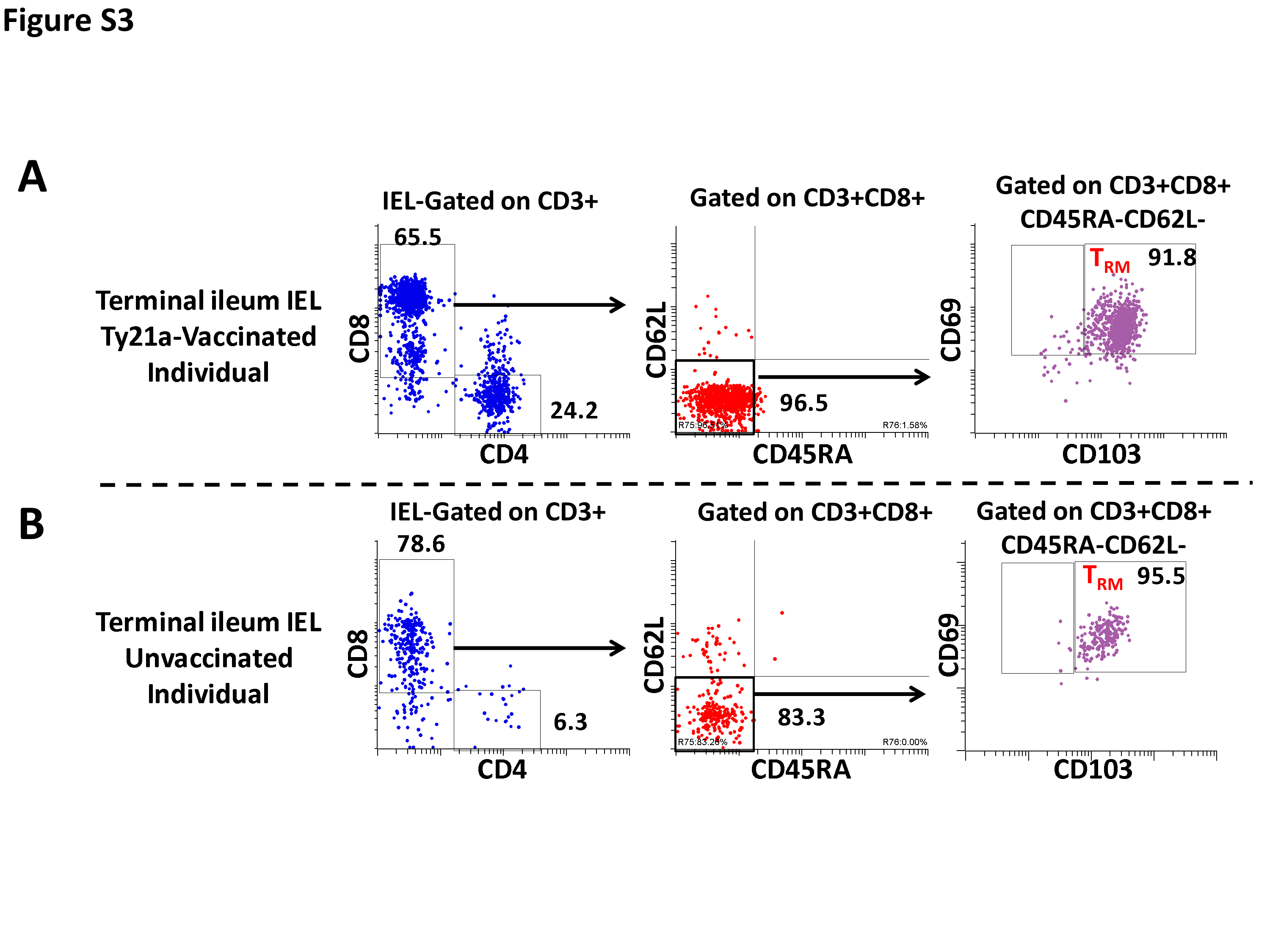

Supplement: Figure S3 — Gating Strategy for identifying terminal ileum IEL CD8+ tissue-resident T memory cell subsets (TRM). Terminal ileum intraepithelial T lymphocytes were isolated and IEL CD8+ tissue resident T memory cell (TRM) identified using CD69 and CD103 markers. Shown are representative volunteers. (A) Ty21a vaccinated. (B) unvaccinated. [file Image_3.TIF]

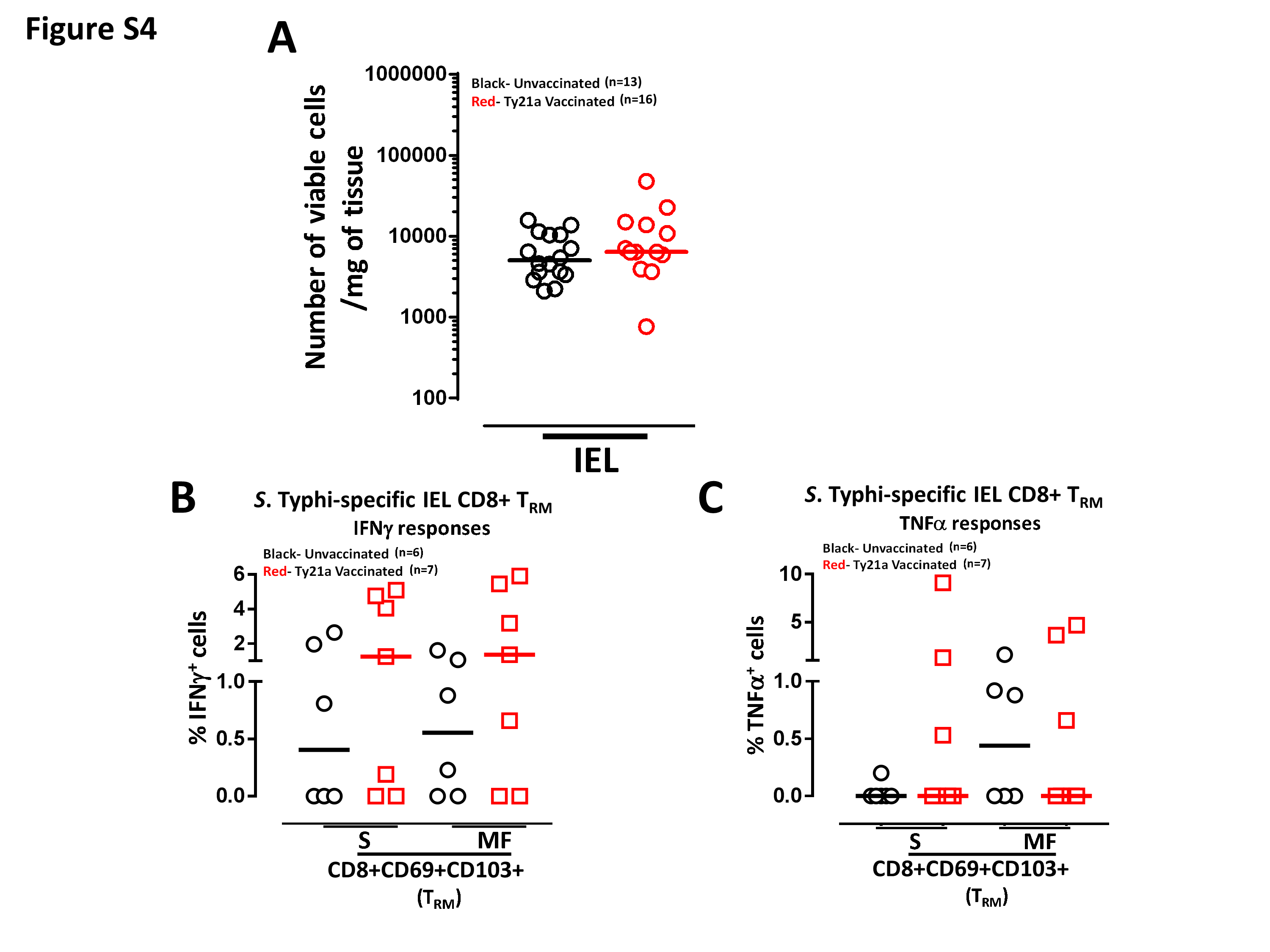

Supplement: Figure S4 — Absolute number of viable terminal ileum cells and S. Typhi-specific responses of terminal ileum IEL CD8+ TRM in healthy adults following oral Ty21a-immunization. (A) The number of freshly isolated terminal ileum IEL obtained from biopsies of Ty21a-vaccinated (n = 13; red symbols) and unvaccinated (n = 16; black symbols) volunteers were compared. The net percentages of S. Typhi-specific responses (IFN-γ, IL-17A, IL-2, and TNF-α) of terminal ileum IEL CD8+ TRM were determined and compared between TI IEL obtained from Ty21a-vaccinated (n = 7; red symbols) and unvaccinated volunteers (n = 6; black symbols). Using the FCOM function of Winlist, IEL CD8+ TRM responses were stratified into multifunctional cells (MF) and single-positive cells (S). Comparison of S. Typhi-specific TI IEL CD8+ TRM responses in (B) IFN-γ+; (C) TNF-α+ Horizontal bars (black and red) represent median values. [file Image_4.TIF]

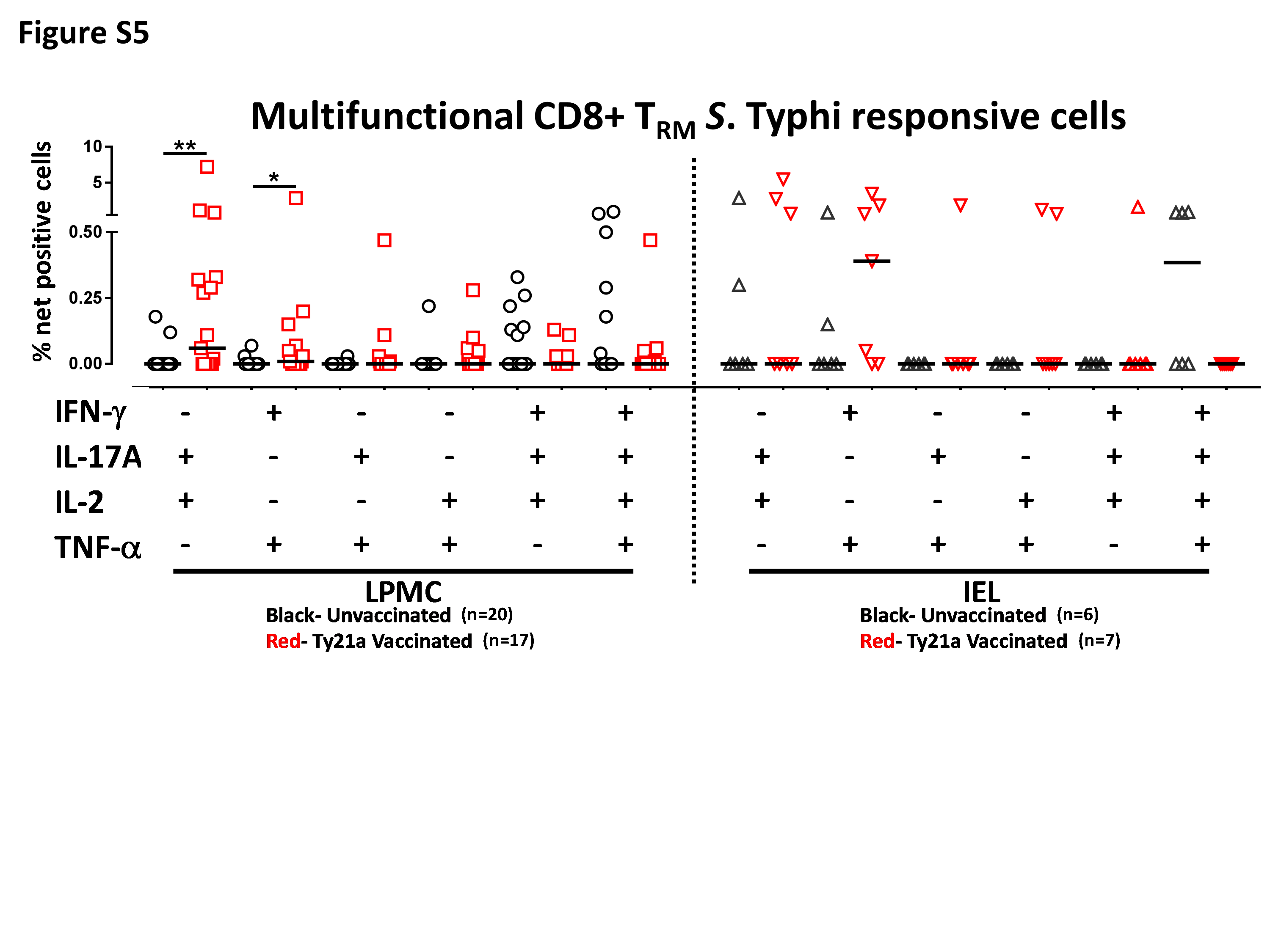

Supplement: Figure S5 — Multifunctional S. Typhi responsive CD8+ TRM responses in terminal ileum LPMC and IEL in Ty21a-vaccinated and unvaccinated volunteers. Induction of multiple cytokines (IFN-γ, IL-17A, IL-2, and TNF-α) in terminal ileum LPMC and IEL following stimulation with S. Typhi-infected EBV-B cells. Data were analyzed using FCOM function of Winlist and six (out of 16) significant combinations are represented in the figure with double, triple and quadruple responses with significant differences (*P < 0.05; **P < 0.005) between vaccinated and unvaccinated volunteers shown. Horizontal bars (black and red) represent median values. [file Image_5.TIF]
