# Supplementary material for: Attenuated Oral Typhoid Vaccine Ty21a Elicits Lamina Propria and Intra-Epithelial Lymphocyte Tissue-Resident Effector Memory CD8 T Responses in the Human Terminal Ileum
Source: Front Immunol. 2019 Mar 14;10:424. doi: 10.3389/fimmu.2019.00424 (PMC6426796; doi:10.3389/fimmu.2019.00424)
Supplement: Table S1 — Demographics of the participants included in this study. [file Table_1.pdf]

**Table S1**

**Table S1. Demographics of the participants included in this study**

| Characteristics | Unvaccinated | Ty21a vaccinated |
|---|---|---|
| Number of volunteers | 20 | 17 |
| Age, mean (range) | 56 (47-73) | 59 (50-73) |
| Sex, # female (%) | 13 (65 %) | 11 (65 %) |
| Ethnicity, # of Caucasian (%) | 11 (55 %) | 11 (65 %) |
| # of African American (%) | 8 (40%) | 5 (29%) |
| # of Asian (%) | 1(5 %) | 1(6 %) |
