# Supplementary material for: Attenuated Oral Typhoid Vaccine Ty21a Elicits Lamina Propria and Intra-Epithelial Lymphocyte Tissue-Resident Effector Memory CD8 T Responses in the Human Terminal Ileum
Source: Front Immunol. 2019 Mar 14;10:424. doi: 10.3389/fimmu.2019.00424 (PMC6426796; doi:10.3389/fimmu.2019.00424)
Supplement: Table S2 — Spearman correlation analysis between LPMC and IEL CD8+TRM S. Typhi responses in unvaccinated and Ty21a-vaccinated volunteers. [file Table_2.pdf]

Table S2

Table S2. Spearman correlation analysis between LPMC and IEL CD8+ T_{RM} *S*. Typhi responses in unvaccinated and Ty21a-vaccinated volunteers

	Terminal ileum LPMC vs IEL (Spearman r)			
	Net *S*. Typhi-specific responses			
	Unvaccinated		Ty21a vaccinated	
	$CD8^+$ T_{RM}- **S**	$CD8^+$ T_{RM}- **MF**	$CD8^+$ T_{RM}- **S**	$CD8^+$ T_{RM}- **MF**
IFNγ	-0.462	0.380	-0.232	0.119
IL-17A	-0.167	0.378	-0.536	**0.817**
IL-2	-0.338	0.102	-0.300	**0.768**
TNFα	-0.133	0.304	-0.155	0.360

Values were not significant unless where indicated. Green - $p<0.005$ **; Red** - $p<0.05$
