# Supplementary material for: Attenuated Oral Typhoid Vaccine Ty21a Elicits Lamina Propria and Intra-Epithelial Lymphocyte Tissue-Resident Effector Memory CD8 T Responses in the Human Terminal Ileum
Source: Front Immunol. 2019 Mar 14;10:424. doi: 10.3389/fimmu.2019.00424 (PMC6426796; doi:10.3389/fimmu.2019.00424)
Supplement: Table S3 — Spearman correlation analysis between mucosal (LPMC and IEL) CD8+ TRM and PBMC TEM S. Typhi responses in Ty21a-vaccinated volunteers. [file Table_3.pdf]

Table S3

Table S3. Spearman correlation analysis between mucosal (LPMC and IEL) CD8+ T_{RM} and PBMC T_{EM} *S.* Typhi responses in Ty21a-vaccinated volunteers

	Ty21a Vaccinated (Spearman r)			
	Net *S* . Typhi-specific responses			
	PBMC T_{EM} vs LPMC T_{RM}		PBMC T_{EM} vs IEL T_{RM}	
	$CD8^+$ T- **S**	$CD8^+$ T- **MF**	$CD8^+$ T- **S**	$CD8^+$ T- **MF**
IFNγ	**0.474**	**0.418**	0.089	-0.038
IL-17A	**0.018**	**0.421**	0.185	-0.075
IL-2	**0.105**	**0.035**	-0.124	-0.661
TNFα	**-0.546**	**0.215**	-0.019	-0.403

Values were not significant unless where indicated. Red -p<0.05
